# Faster embryonic segmentation through elevated Delta-Notch signalling

**DOI:** 10.1038/ncomms11861

**Published:** 2016-06-15

**Authors:** Bo-Kai Liao, David J. Jörg, Andrew C. Oates

**Affiliations:** 1 Max Planck Institute of Molecular Cell Biology and Genetics, Pfotenhauerstr. 108, Dresden 01037, Germany; 2 Francis Crick Institute, Mill Hill Laboratory, The Ridgeway, London NW7 1AA, UK; 3 Max Planck Institute for the Physics of Complex Systems, Nöthnitzer Str. 38, Dresden 01187, Germany; 4 Department of Cell and Developmental Biology, University College London, Gower Street, London WC1E 6BT, UK

## Abstract

An important step in understanding biological rhythms is the control of period. A multicellular, rhythmic patterning system termed the segmentation clock is thought to govern the sequential production of the vertebrate embryo's body segments, the somites. Several genetic loss-of-function conditions, including the Delta-Notch intercellular signalling mutants, result in slower segmentation. Here, we generate DeltaD transgenic zebrafish lines with a range of copy numbers and correspondingly increased signalling levels, and observe faster segmentation. The highest-expressing line shows an altered oscillating gene expression wave pattern and shortened segmentation period, producing embryos with more, shorter body segments. Our results reveal surprising differences in how Notch signalling strength is quantitatively interpreted in different organ systems, and suggest a role for intercellular communication in regulating the output period of the segmentation clock by altering its spatial pattern.

## Abstract (web summary)

Rhythmic patterning governs the formation of somites in vertebrates, but how the period of such rhythms can be changed is unclear. Here, the authors generate a genetic model in zebrafish to increase DeltaD expression, which increases the range of Delta-Notch signalling, causing faster segmentation.

## Graphical abstract

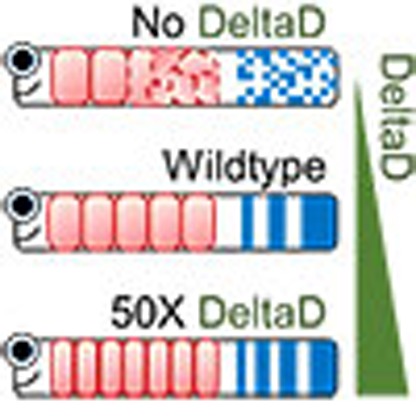

The temporal coordination of events is an important, yet poorly understood aspect of development. A valuable model to investigate developmental timing is the rhythmic and sequential segmentation of the embryonic body axis into a series of multicellular units that anticipate the adult form. In vertebrates, these are called somites and give rise to segmented anatomy of the adult, such as vertebra, ribs and the associated muscles and skin. The rhythmic formation of embryonic segments is thought to be governed by a multicellular system of oscillating gene expression, termed as the ‘segmentation clock', present in the posterior unsegmented tissue, the tailbud and presomitic mesoderm (PSM)^1^. Waves of gene expression appear in the posterior of the tissue and travel to the anterior where their arrest prefigures the time and position of each newly forming segment. These waves emerge at the tissue level from the coordinated output of cellular genetic oscillators, and their wavelength decreases as they move anteriorly. The period with which new segments are formed depends on the time scales of the genetic oscillations, and recent findings in zebrafish show that the period of segmentation can also be influenced by features of the wave pattern^2^. Despite this emerging picture, the genetic mechanisms that control the period at these various levels are not well-understood.

Although loss-of-function mutants in several genes increase the period of segmentation^3^, manipulating the system to run faster has been difficult. Removing introns from the mouse *Hes7* gene leads to faster segmentation of the anterior axis, but the altered segmentation clock is unstable and segmentation is defective posterior to the neck^4^. Faster and stable segmentation of the entire body axis has not been engineered.

A central genetic player in segmentation is the Delta-Notch intercellular signalling pathway. The pathway has important roles in patterning and differentiation of many organ systems across the animal kingdom, and was associated with the rhythms of segmentation because of the waves of gene expression of several pathway members in the PSM^5^. In the zebrafish, the Delta-Notch pathway has been implicated in the synchronization of neighbouring oscillating PSM cells, leading to a coherent rhythm at the tissue level^6^,^7^,^8^,^9^,^10^,^11^,^12^. There are two *delta* genes expressed in the zebrafish PSM. Although *deltaC* shows oscillatory waves of expression and *deltaD* does not^8^, the formation of heterodimeric complexes between DeltaD and DeltaC^13^ and their joint regulation by the ubiquitin ligase Mind bomb^14^,^15^ suggest that both ligands contribute to rhythmic signalling between neighbouring PSM cells. *deltaD* mutants, known as *after eight* (*aei*)^16^,^17^, form their first 10 segments ∼20% slower than wildtype (WT) and the length of these segments is correspondingly larger; other loss-of-function mutations and pharmacological treatments affecting this pathway show a similar phenotype^18^. Early during this developmental interval the wave pattern has a longer wavelength in the anterior PSM in *mind bomb* mutants than in WT siblings. In all loss-of-function cases, the coherent wave pattern and segment boundary formation is finally disrupted by the loss of synchrony leading to segmentation defects in the posterior trunk and the tail^8^,^10^,^11^,^18^.

These results raise the possibility that an increase in Delta-Notch signalling may shorten the period of segmentation. However, previous over-expression studies using mRNA injection or ubiquitous heat-shock induction of Delta and Notch resulted in segmentation defects^10^,^12^,^19^,^20^,^21^. The lack of the endogenous spatiotemporal expression pattern in these over-expression approaches may have masked a potential activity on the period of segmentation. In this report, we ask whether an appropriately directed increase in Delta-Notch signalling causes a change in segmentation period. We approach this by engineering zebrafish with extra copies of a transgene containing the *deltaD* locus within its full genomic regulatory region to drive the correct spatiotemporal expression pattern. We assay protein levels, neurogenesis and segmentation in a set of DeltaD transgenic lines with different copy number and find that neurogenesis is sensitive to small changes in DeltaD levels, whereas only the highest level of DeltaD expression results in a shorter segmentation period. This shorter segmentation period is explained by elevated coupling between oscillators leading to changes in the synchronized wave pattern. Thus, beyond its previously described role in synchronizing oscillators, these findings suggest that Delta-Notch signalling can also affect the wavelength of the tissue's spatial pattern and thereby alter its output period.

## Results

### Transgenic lines with elevated accurate DeltaD expression

To obtain accurate spatiotemporal expression, we used BAC recombineering to replace the stop codon of the *deltaD* gene with *venus* yellow fluorescent protein (YFP) in a 14-Kbp piece of genomic DNA that encompassed the *deltaD* exons^22^ (Supplementary Fig. 1). The *venus*-YFP tag was inserted to facilitate cellular-level imaging of DeltaD protein *in vivo*, which will be reported elsewhere; here we focus on the phenotypic effects of this transgene. We generated several *deltaD* transgenic lines using I-SceI meganuclease-mediated transgenesis^23^ to get single insertion-site, variable copy number lines in the *aei*/*deltaD* mutant background.

**Figure 1:**
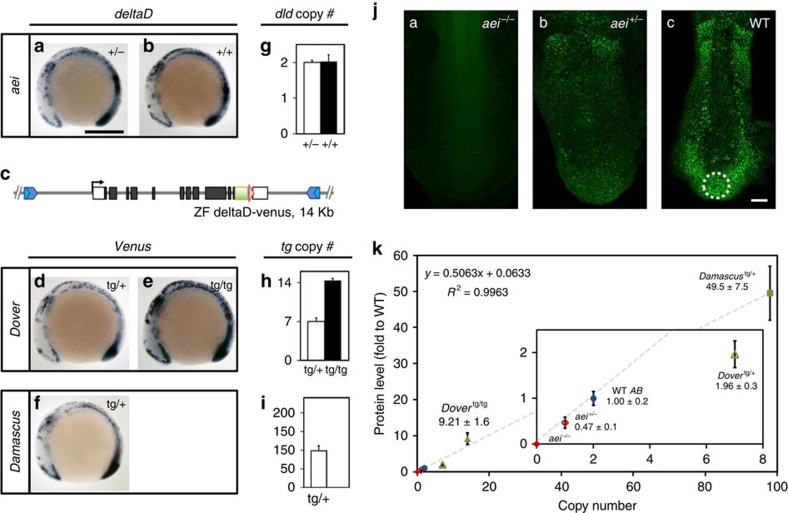
A range of transgenic lines with elevated spatiotemporally accurate DeltaD expression. (**a**–**f**) *deltaD-venus* recapitulates WT *deltaD* expression pattern. Whole-mount ISH, 9 somite-stage embryos lateral view with either *deltaD* or *egfp* anti-sense riboprobe in *deltaD* mutant *after eight* (*aei*)^+/−^ (**a**), WT *AB* (**b**) or *deltaD-venus* transgenic lines (**d**–**f**). (**c**) Schematic representation of *deltaD-venus* construct for generating transgenic lines. See Supplementary Fig. 1. (**a**) Scale bar, 300 μm. (**f**–**h**) *deltaD* transgenic copy numbers determined by quantitative real-time PCR from genomic DNA. Copy number is insensitive to *aei* point mutation allele (**g**). Transgenic copies (**h**,**i**) are total *deltaD* copies minus 2 endogenous copies. Empty bars, heterozygous genotypes; black bars, homozygous genotypes. Data pooled from ⩾3 independent experiments, mean±s.d. (**j**) DeltaD protein expression visualized and quantitated by immunostaining. (ja–jc) PSM of flat-mounted 9 somite-stage embryos, circle shows region used for intensity measurement. Scale bar, 50 μm. (**k**) DeltaD protein expression levels versus *deltaD* gene copy number. Expression level in *aei*^−/−^ was defined as 0. Grey dashed line, linear fit as the formula and *R*^2^ value at upper left corner. Inset shows magnification of data points at origin. Mean±s.d. (*n*⩾5).

We analyzed two lines that we named *Dover* and *Damascus*. The mRNA patterns from the transgenic loci are indistinguishable from WT *deltaD* expression (Fig. 1a–f). Staining intensity from the homozygous *Dover* was higher than the heterozygote (Fig. 1d,e). We measured the copy number of *deltaD*-*venus* transgenes by Q-PCR and found that *Dover* has 7 copies and *Damascus* has approximately 100 (Fig. 1g–i). We examined DeltaD protein levels by quantitation of whole-mount immunohistochemistry using monoclonal DeltaD antibodies^13^. We were able to quantitatively distinguish between WT, heterozygote and homozygote *aei/deltaD* mutants (Fig. 1j, inset of k). We observed an increased level of DeltaD in the tailbud and PSM in the transgenic lines ranging from a twofold increase in a *Dover* heterozygote up to a 50-fold increase in the *Damascus* heterozygote (Fig. 1k). We conclude that we made transgenic zebrafish with a range up to 50-fold excess of DeltaD, and with apparently normal expression patterns.

### Elevated DeltaD shortens segmentation period

**Figure 2:**
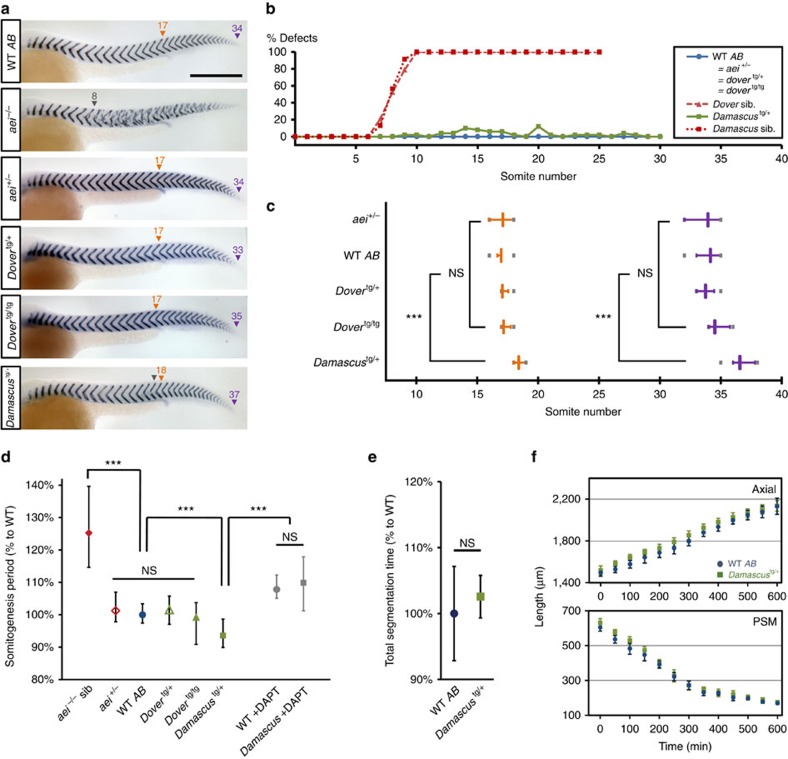
Elevated DeltaD speeds somitogenesis. (**a**) Representative embryos at 36 h post fertilization (h.p.f.) stained with segment boundary marker *cb1045* (*xirp2a*). Rescue of segmentation defects in *aei*^−/−^ genotype background by *dover* and *damascus*. Numbers above arrowheads indicate the posterior boundaries of the corresponding ordinal segments. Orange triangle, segment boundary aligned with proctodeum; purple triangle, last segment boundary observed; grey triangle, defective segment boundaries or onset of defects. Scale bar=300 μm. (**b**) Distribution of somite defects showing *deltaD-venus* rescues *aei*^−/−^ throughout somitogenesis. No defective boundaries observed in WT *AB* (*n*=74), *aei*^+/−^ (*n*=76), *Dover*^tg/+^ (*n*=95) and *Dover*^tg/tg^ (*n*=60) (blue line). *Damascus* showed low penetrance of defects (2.6% from the 1st to 30th segments, *n*=64) (green line). *aei*^−/−^ siblings (sib) of *dover* (*n*=29) or *Damascus* (*n*=33) showed typical *deltaD* lost-of-function phenotypes, mean ALD around eight (red lines). (**c**) *Damascus* has more segments and shifted segmental position of proctodeum. Quantification from embryos as shown in **a**. Orange, level of proctodeum; magenta, total segment number; grey bars, extreme values. (**d**) Somitogenesis period of trunk region (s4–s19) measured at 28.3±0.3 °C by multiple embryo time-lapse imaging. Mean periods normalized to WT *AB*. Data pooled from ⩾3 independent trials (*n*⩾12 embryos for each condition), except *aei*^−/−^ siblings from 1 trial (*n*=6). (**e**) Total segmentation time is the interval between the 4th segment to the last segment observed in time-lapse movies. Quantification of corresponding movies described in **b**. Data pooled from ⩾3 independent trials (*n*⩾12 embryos for each condition). Mean durations normalized to WT *AB* (**f**) Axis elongation and PSM shortening are not altered in *Damascus*. Data analyzed from same time-lapse movies as **d**. Time 0 is defined at 5 ss. ***P≤0.001, NS, not significant (*P*>0.05), Student's *t*-test. Error bars show the 5th and 95th percentiles of the data.

Before we assessed the transgenes for an effect on the period of the segmentation clock, we first tested them for activity by their ability to rescue the fully penetrant *aei/deltaD*^−/−^ segmentation phenotype. Using a high-contrast stain for *xirp2a* mRNA to detect the segmental boundaries, both *Dover* and *Damascus* rescued (Fig. 2a,b). *Damascus* had a low frequency (<3%) of segment defects scattered along the axis. We counted the total number of segments made in the body (Fig. 2c). Although the total duration of segmentation was the same (Fig. 2e), *Damascus* made 7.6% more total-body segments (WT 34.1±0.7 mean±s.d., *n*=34; *Damascus* 36.7±0.7, *n*=25; *t*-test *P*<0.001), and made 8.2% more segments in the trunk than the WT (WT 17.0±0.3, *n*=34; *Damascus* 18.4±0.5, *n*=25, *t*-test *P*<0.001), indicating that the extra segments were not added to the posterior tail. The other transgenes had no significant effect (*t*-test *P*>0.05).

We measured the segmentation period directly by multiple embryo time-lapse microscopy (Fig. 2d). *Damascus* made its (trunk) somites faster than WT siblings, with segmentation periods of 23.1±0.8 and 24.7±0.6 min, respectively (wildtype *n*=26; *Damascus n*=12; *t*-test *P*<0.001). The magnitude of the decrease in period, 6.5%, is consistent with the increase in trunk segment number and total-body segment numbers. The effect on period was lost by incubating *Damascus* embryos in N-[N-(3,5-difluorophenacetyl)-L-alanyl]-S-phenylglycine t-butyl ester (DAPT), which blocks the γ-secretase cleavage of the Notch intracellular domain^24^. This suggests that an elevated Notch signalling level is responsible for the shorter segmentation period. We conclude that the *Damascus* transgenic line has a decreased segmentation period and makes more, shorter segments.

### Altered gene expression patterns linked to period change

Recent work suggests that the wave pattern of oscillating gene expression in the PSM contributes to the period of segmentation in a shortening tissue due to a Doppler effect^2^. This Doppler effect arises because, as the tissue shortens, the anterior end of the PSM where the new segments are generated moves into the waves of oscillating gene expression and hence acts like an observer moving towards a wave-emitting source. This movement locally decreases the observed period with which waves arrive at the anterior end relative to the period of genetic oscillations in the posterior PSM. Since each wave arriving at the anterior end corresponds to one newly forming segment, this Doppler effect mediates a decrease in the period of segment formation. The magnitude of the Doppler effect is determined by the velocity of tissue shortening and the wavelength at the anterior end^2^,^25^ (Supplementary Note 1).

**Figure 3:**
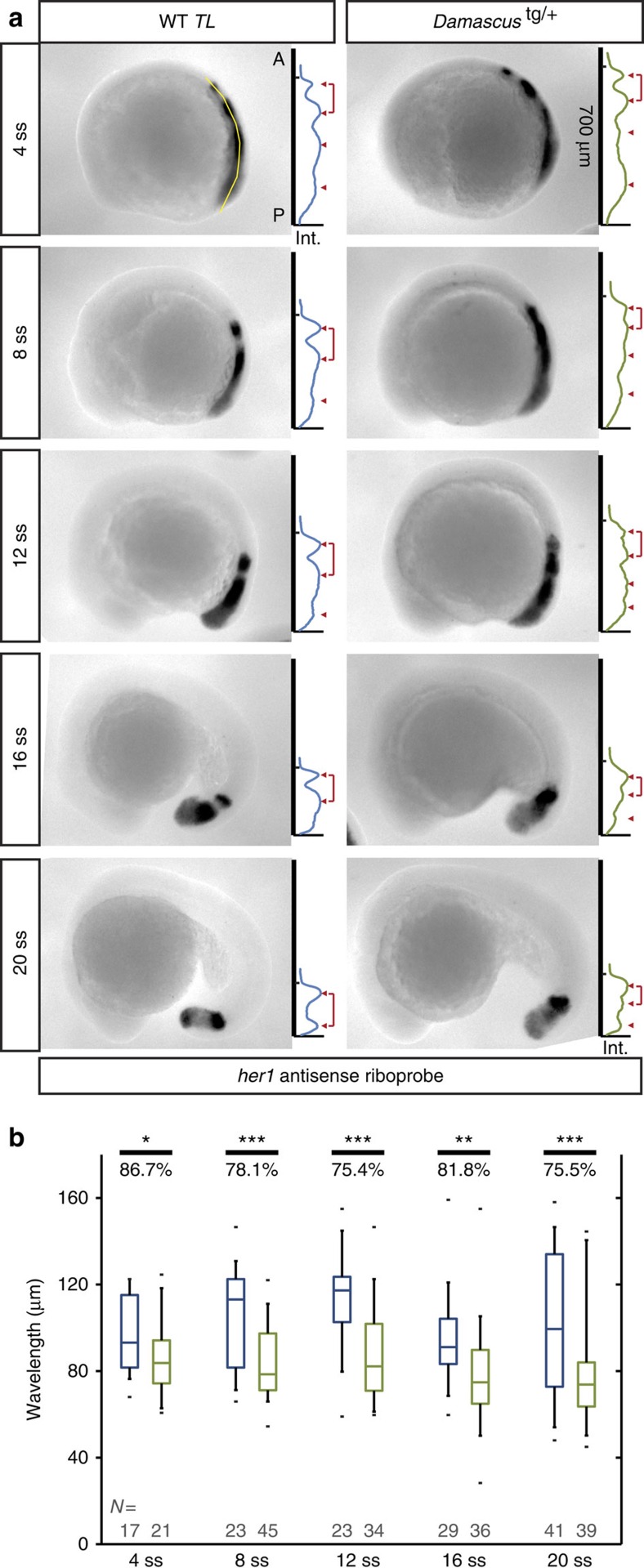
*Damascus* has shorter anterior wavelength. (**a**) Measurement of *her1* anterior wavelength from 4 to 20 somite-stage. In each representative embryo panel, left part: examples of WT *TL* or *Damascus*^tg/+^ embryos in lateral view, anterior to left, after ISH with *her1* probe. The line of interest for image processing is visible in yellow, extending from the tip of the tail bud to the somites. Right part: (inverted) gene expression signal intensity (*x* axis) versus position along the anterior-posterior axis (*y* axis) plots for the embryos in the left-hand part. The tick marker on the *y* axis indicates the *X*_0_ position, and *y* axis is the scale bar (700 μm) to its representative embryo as well. Peak maxima is indicated with red triangles. The anterior wavelength, defined as the distance between the most anterior two gene expression maxima, is indicated by a red bracket. A, anterior; Int., intensity in arbitrary unit; P, posterior. (**b**) The most anterior wavelength of *her1* mRNA gene expression pattern. The average ratio (in percentage) between WT *TL* or *Damascus*^tg/+^ embryos for each stage examined are labelled above the plots. Blue, WT; Green, *Damascus*^tg/+^. The central boxes of box-and-whisker plots cover the interquartile range with median as line within box. Whiskers are 5th and 95th percentiles, extreme values are grey bars. *P≤0.05, **P≤0.01, ***P≤0.001, Student's *t*-test.

To test the hypothesis that a change in the Doppler effect contributes to the change in segmentation period in *Damascus*, we measured tissue-length change and anterior wavelength of the gene expression pattern. The rate of extension of the body axis and rate of shortening of the PSM was not different in *Damascus* and WT (WT axial extension 1.13±0.05 μm min^−1^, *Damascus* 1.06±0.11 μm min^−1^, *t*-test *P*=0.154; WT PSM shortening 0.80±0.07 μm min^−1^, *Damascus* 0.79±0.03 μm min^−1^, *t*-test *P*=0.791; rate determined from the linear fit from 0 to 600 min) (Fig. 2f), ruling out a contribution from a change in the velocity of tissue shortening. In contrast, we observed a systematically shorter anterior wavelength in the wave pattern of endogenous *her1* expression in *Damascus* compared to WT across the developmental interval encompassing trunk segmentation (Fig. 3a,b; Supplementary Fig. 2). Features of the wave pattern were also changed more posteriorly in the PSM, as evidenced by an increase in the number of gene expression waves observed in *Damascus* (Fig. 3; Supplementary Fig. 2). Thus, elevated DeltaD expression creates a characteristic new wave pattern in *Damascus*.

To assess the magnitude of the Doppler effect, only the anterior-most stripe is relevant and from the contribution of this change in the wave pattern, we calculated the expected period in *Damascus* to be 23.3±0.3, 5.7% shorter than WT (see Methods, Supplementary Note 2; Supplementary Table 1; Supplementary Fig. 3). This accounts for the majority of the observed 6.5% difference in segmentation period in this interval (Fig. 2d). Combined, these results fulfil the expectation of a ‘segmentation clock' with a shorter output period in an otherwise normally growing embryo.

Why does elevated DeltaD expression lead to an altered wave pattern with a shorter anterior wavelength in *Damascus*? Elevated Delta expression would be anticipated to cause elevated Notch signalling, and this in turn would lead to an elevated coupling between neighbouring oscillators^8^,^11^. Previous theoretical work has suggested that altered coupling can alter the spatial pattern and output period of a ‘segmentation clock'^18^,^26^,^27^, and we return to examine this process in the last section of the results.

Interpreting the period phenotype requires a better understanding of the relationship between Delta expression levels and Notch signalling. We expect that in the various lines we have generated (Fig. 1j,k), increased levels of DeltaD protein expression give rise to correspondingly elevated levels of Delta-Notch coupling between neighbouring cells. However, the lack of an effect on the period in *Dover* raises the possibility that Notch signalling was not elevated in this line. Alternatively, the *Damascus* allele may have a neomorphic activity. A third possibility is that the segmentation clock is strongly buffered against increases to Delta-Notch signalling. To investigate this further, we examined two indexes of Notch signalling in the embryo, lateral inhibition and synchronization, across the range of DeltaD expression levels.

### Elevated DeltaD increases lateral inhibition in the CNS

Firstly, we tested whether the transgenic lines had an effect on primary neurogenesis. The role of Delta-Notch signalling in neurogenesis is better understood than in somitogenesis, and is thought to involve a process termed lateral inhibition that generates fine-grained patterns in which neighbouring cells take on differing fates^28^,^29^,^30^. Elevated Delta levels, and thus increased Notch activation, yields a reduced number of cells selecting the primary neuronal fate in all species investigated^31^,^32^,^33^,^34^.

**Figure 4:**
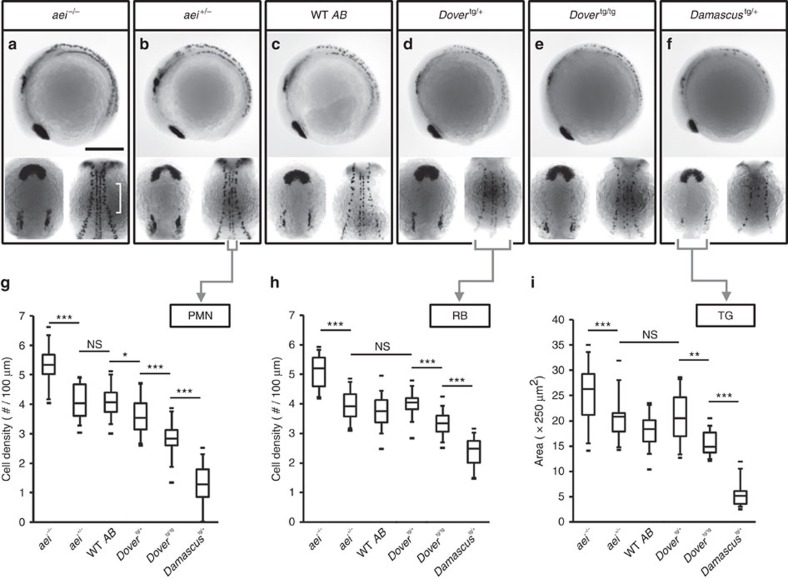
Elevated DeltaD increases the strength of lateral inhibition in the CNS. (**a**–**f**) Representative embryos stained with *islet1*, an early marker of primary neuronal differentiation. In each box, 4 somite-stage embryos lateral view, anterior to left (upper panel); dorsal view with anterior to top (lower left panel), of head showing bilateral trigeminal ganglia (TG); mid-trunk showing PMN at the midline, and RB neurons at lateral margin of neural plate (lower right panel). (**a**) Scale bars, 250 μm. (**g**) Cell density of PMN. (**h**) Cell density of RB sensory neurons. (**i**) Area of the TG. Lower densities or smaller areas indicate higher Notch signalling strength. Data from *aei*^−/−^ (*n*=33), *aei*^+/−^ (*n*=18), WT (*n*=35), *Dover*^tg/+^ (*n*=14), *Dover*^tg/tg^ (*n*=12) and *Damascus*^tg/+^ (*n*=22) pooled from ⩾2 independent trials. The central boxes of the box-and-whisker plots cover the interquartile range with median as line within box. Whiskers are 5th and 95th percentiles, extreme values are small bars. *P≤0.05, **P≤0.01, ***P≤0.001, NS, not significant (*P*>0.05), Student's *t*-test.

We examined the numbers of primary motor neurons (PMN), Rohan–Beard neurons (RB) and trigeminal neurons (TG) by *islet1* expression^35^. For each subtype, *aei/deltaD* homozygote embryos had more primary neurons than WT (Fig. 4a,g–i), as expected^16^,^22^,^34^. With higher copy number of transgenes, we found a graded decrease in the density of neurons for all categories (Fig. 4d–i). Primary motor neurons were the most sensitive to changes in *deltaD* copy number: the embryo distinguished between all copy number differences except between 1 and 2 (Fig. 4g). This shows that the *Dover* transgene produced elevated levels of active Delta and this translated into correspondingly elevated Notch signalling. It also indicates that *Damascus* acts as a strong hypermorphic allele of *deltaD* in the nervous system. These results suggest that, in contrast to Delta's effect on segmentation period, the lateral inhibition mechanism in the CNS converts the input level of Delta protein smoothly to the Notch signalling output.

### Elevated DeltaD increases coupling between PSM oscillators

**Figure 5:**
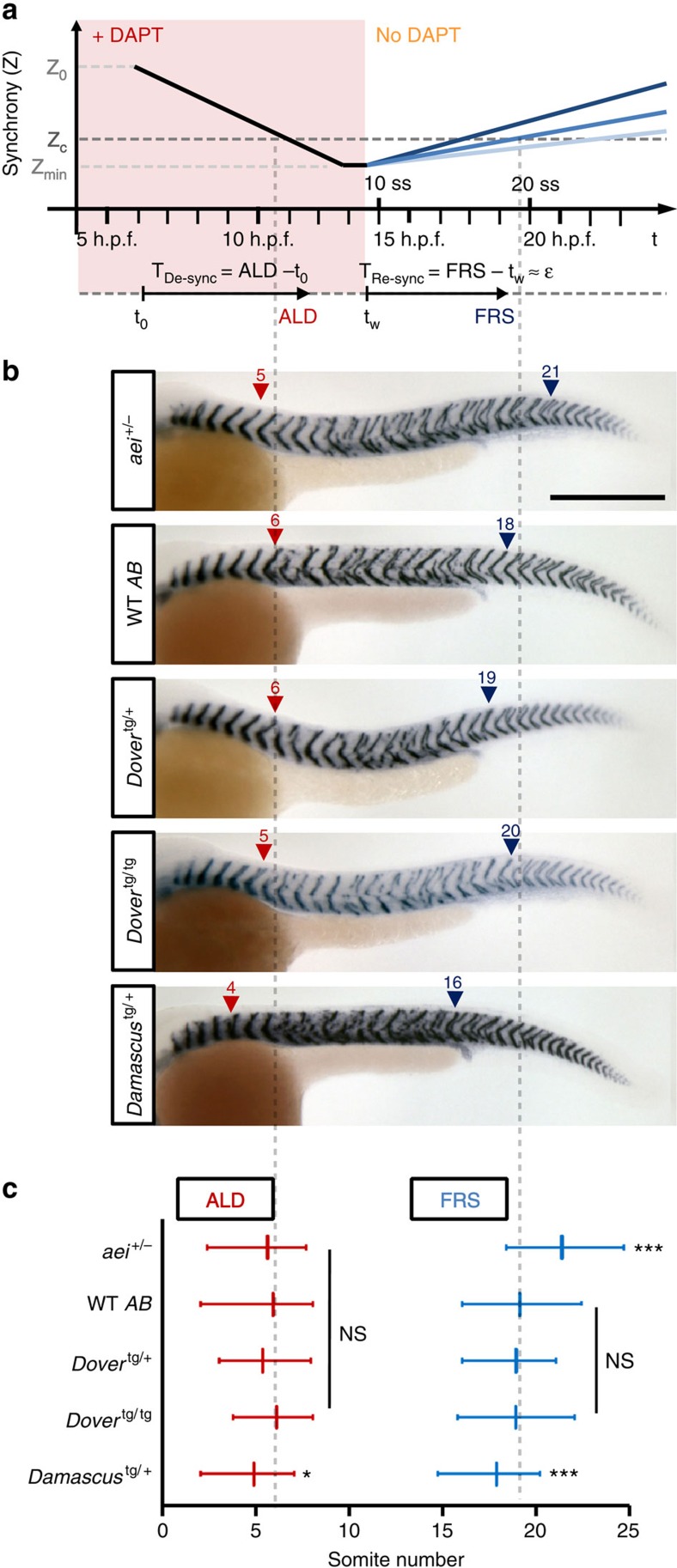
Elevated DeltaD increases the strength of coupling in the segmentation clock. (**a**) Schematic of DAPT pulse-chase synchrony rescue assay for estimating relative coupling strength. Presumptive synchrony level (*Z*) versus experimental time scale shown in upper panel. *Z* is an order parameter that measures the degree of synchrony between neighbouring oscillators, with 1 indicating perfect synchrony and 0 indicating complete lack of temporal correlation, as defined in ref. 11. *Z*_0_, initial synchrony level at shield stage (6 h.p.f.); *Z*_c_, critical level of synchrony for forming non-defective segment boundaries; *Z*_min_, putative minimal synchrony at de-synchronized state; darkness of blue lines depict relative coupling strength. Time intervals (*T*_Re-sync_) between DAPT wash-out and resynchronized states reflect coupling strength (ɛ). (**b**) Segment boundaries after DAPT pulse-chase in mutant and transgenic backgrounds. Numbers above arrowheads indicate the values of ALD (red triangle) or FRS (blue triangle). Scale bar, 300 μm. (**c**) Quantification of DAPT pulse-chase experiments. ALD (red) and FRS (blue) from left or right side independently, as shown in **a**. Data from *aei*^+/−^ (*n*=48), WT (*n*=94), *Dover*^tg/+^ (*n*=43), *Dover*^tg/tg^ (*n*=36) and *Damascus*^tg/+^ (*n*=78) pooled from three independent trials. Data is mean value with error bars showing 5th and 95th percentiles. *P≤0.05, ***P≤0.001, NS, not significant (*P*>0.05), Student's *t*-test.

Secondly, we tested whether Notch signalling was elevated in the segmenting tissue using a modified, quantitative re-synchronization assay^9^,^11^ (Fig. 5a). In this assay, Notch signalling is blocked before the onset of oscillations in the early embryo by incubation in saturating DAPT, which prevents proteolytic cleavage of the intracellular domain. Over time, the population of cellular oscillators desynchronizes at a rate set by the noise level of the individual oscillators, until falling below a critical level where penetrant segmentation boundary defects are observed posterior to approximately segment number 5, the anterior limit of defects (ALD)^8^,^11^. When DAPT is washed out, Notch signalling begins to couple the neighbouring oscillators, and the population gradually resynchronizes. The position, and hence time, of the first segment boundary to form correctly again is recorded as a proxy for the synchronization state of the population having risen beyond the critical level. We term this the first recovered segment (FRS). The time taken to re-synchronize is expected to depend on the coupling strength^11^, and faster re-synchronization, that is, lower FRS, is expected from a higher Delta-Notch coupling strength.

We asked whether re-synchronization was sensitive to the difference between one and two functional copies of *deltaD*, and observed a higher FRS in *aei/deltaD* heterozygotes than WT (WT 19.1±2.2, *n*=94; *aei*^+/^^−^ 21.3±2.0, *n*=48; *t*-test *P*<0.001), indicating a 20.5% slower re-synchronization time (Fig. 5b,c). We measured the re-synchronization time in the transgenic lines and found that *Damascus* had a lower FRS than WT and *Dover* (*Damascus* 17.8±1.9, *n*=78; *t*-test to WT *P*=0.002), indicating that *Damascus* rescued segment boundaries 15.0% faster (Fig. 5b,c). Although we cannot rule out subtle changes to the intracellular oscillator circuit, for example, a potential increase in the noise as evidenced by a decrease in ALD (Fig. 5c), this faster re-synchronization can be explained by a higher coupling strength in *Damascus*, consistent with elevated Notch signalling between oscillators in the population. Because the re-synchronization time did not change in *Dover*, these results suggest that the synchronization mechanism is strongly buffered across a range of Notch signalling.

### Wave patterns in a simplified model of coupling with delays

In systems of coupled oscillators, time delays in the coupling can give rise to a plethora of complex phenomena. In considering these effects, it is important to distinguish between the strength of coupling and its delay. Coupling strength can be thought of as the number of activated ligand–receptor pairs over time, which is expected to increase as a consequence of the over-expression of DeltaD. In contrast, the time taken to transfer this signal from one cell to another is termed the coupling delay. Because of the time required to synthesize and traffic Delta proteins, a non-negligible time delay in the coupling is expected for oscillators coupled by the Delta-Notch signalling^13^,^18^,^36^. Direct measurement of coupling strength and time delays is not currently possible in any embryo, although values for WT and mutant zebrafish have been inferred^18^. To investigate whether an internally consistent description of the spatial features of the wave patterns observed in *Damascus* can be found in the presence of elevated coupling strength and realistic time delays, we turned to a simplified theoretical description, which ignores the effects of tissue shortening^26^.

We numerically calculated wave patterns of gene expression for the zebrafish system using a range of coupling strengths and time delays (Supplementary Note 3). We observed wave patterns with features corresponding to the WT embryo, as expected, as well as wave patterns corresponding to *Damascus*. These latter resulted from elevated coupling strength and a plausible alteration of the coupling delay (Supplementary Fig. 4). Supporting the robustness of this altered state to parameter changes, we found multiple regions in parameter space in which elevated coupling strength gives rise to a stable time-periodic wave pattern with an increase in the number of waves and a shortened anterior wavelength (Supplementary Fig. 5) as observed experimentally in *Damascus*. These theoretical results provide an internally consistent description that qualitatively describes the differences between the phenotypes of WT and *Damascus* embryos, supporting the interpretation of an elevated coupling strength in *Damascus*.

## Discussion

In this report, we describe the first embryo genetically engineered to tick more quickly throughout development. This was achieved by the generation of a zebrafish line carrying multiple copies of a YFP-tagged *deltaD* transgene expressed from within its endogenous flanking genomic DNA. Direct measurements of a shorter segmentation period and a corresponding increase in the total number of segments in an otherwise normally-developing embryonic axis argue strongly for a ‘segmentation clock' with a short output period.

A change in segmentation period in an animal with altered Delta/Notch signalling could potentially result from the effects of coupling on the overall period of a population of oscillators, if the coupling has time delays on the order of the period, as is thought to be the case in vertebrate embryos^27^. A lengthening of period due to this process has been inferred for embryos with a reduction in Delta-Notch signalling in the zebrafish^18^. In contrast, mouse segmentation appears to get faster with pharmacological blockade of Notch^37^, an outcome predicted if the length of the time delay in the coupling was shorter than half the period^27^. These descriptions of the system, as used in Supplementary Figs 4 and 5 to check for the effect of an elevated coupling strength, are based on a simplified scenario with a time-periodic wave pattern and constant tissue length.

Recent findings of the effects of waves of gene expression and shortening tissue on segmentation period raise the possibility that changes to the normal pattern of the waves could alter the segmentation period^2^. Our results show that *Damascus* embryos have a shorter gene expression wavelength in the anterior PSM, and the magnitude of this change accounts for most of the observed change in segmentation period through a change in the contribution of the Doppler effect. Regulation of the wave patterns has been proposed to result from gradients of signalling that span the PSM, which are generated by elevated levels of Fgf and Wnt in the posterior^27^,^38^,^39^,^40^,^41^,^42^, but theoretical work shows that changes in the coupling could also alter the wave patterns^18^,^26^,^27^,^43^.

Our observations of elevated Delta-Notch signalling throughout the embryo, including a change in re-synchronization rate, imply that a change in cell–cell communication is responsible for the shorter anterior wavelength. These effects seem likely to be caused by an elevation in Delta-Notch signalling strength between neighbouring oscillators, but we cannot rule out a contribution from changes to signalling delays, in addition. Combining these lines of evidence, this finding supports the prediction that the changed wave pattern results in a change in the magnitude of the Doppler effect. Since one arriving wave at the anterior end is related to one newly formed segment, this wave effect corresponds to a shortening of the period of segment formation.

Direct observation of the genetic oscillations is not currently possible in *Damascus* because available reporters of the segmentation clock in zebrafish express *Venus*-YFP^2^,^6^, as does *Damascus*. However, future experiments will investigate whether the observed changes to the anterior wavelength are accompanied by changes to the period of genetic oscillations in the tailbud, or longer-term changes in the wave pattern^2^,^25^. This will provide a fuller picture of the effects of elevated DeltaD signalling in zebrafish segmentation. Our results reinforce the idea that the period of somitogenesis is influenced by several different processes: by single-cell *Hes*/*Her* gene feedback circuits^4^,^44^,^45^, multicellular local Delta-Notch signalling^18^,^37^, and by the global patterns of cyclic gene expression waves and changing tissue length^2^,^25^.

**Figure 6:**
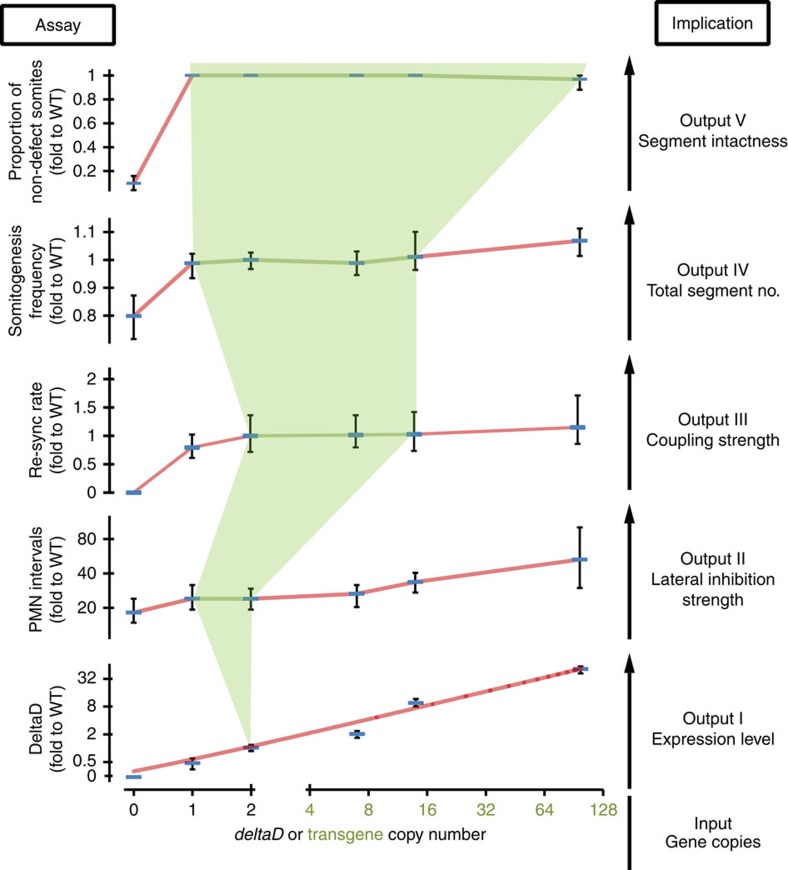
Robustness and flexibility of Delta-Notch signalling. Five experimentally measured system outputs (Assay, left-had axes) and their corresponding biological meaning (Implication, right-hand) are displayed as functions of *deltaD* copy numbers, shown on *x* axis in log_2_ scale, the 0.5 position is defined as 0 copy for *aei*^−/−^. DeltaD protein levels in posterior PSM from Fig. 1k shown in log_2_ scale, the 0.125 position on the axis defined as 0. PMN intervals from Fig. 4g. Re-synchronization (re-sync) rate calculated from the reciprocal of re-synchronization time from DAPT pulse-chase experiments (Fig. 5c), value of *aei*^−/−^ is defined as 0. Somitogenesis frequency from Fig. 2d. Proportion of non-defective somites from Fig. 2b. Lines are connections of data points except for DeltaD protein levels, which is linear fit. The five outputs were ordered from bottom to top based on presumptive increasing robustness of the system. The light green background marks the robust zone, where copy number does not affect system output, and the outer areas reflect the flexibility of output based on the change of WT and transgenic *deltaD* copy numbers. Data is mean value with error bars showing 5th and 95th percentiles. Green lines connect data points that are not significant (*P*>0.05) to WT in Student's *t*-test.

Delta-Notch signalling is essential for multiple events in development, coordinating patterning and differentiation in space and time in various organ systems. It is not well-understood how similar signals are used to different effect in different tissues, for example, synchronization versus lateral inhibition, nor how such differences evolve. We observed a striking difference in how a change in gene copy number was interpreted in segmentation compared to neurogenesis. Segmentation is sensitive to changes in coupling strength in the range from 0 to 2 copies of WT Delta or Notch genes (this study and refs 9, 11), but its properties appear to be well buffered for increased Notch signalling strength until much higher levels are attained (Fig. 6). In contrast, neurogenesis appears to be sensitive to changes in Notch signalling strength across the full range of endogenous and transgenic copy numbers in this study.

Given the frequency of gene duplication observed in metazoan lineages^46^,^47^, the range of copy numbers we assayed may mimic a relatively common mutational scenario. From our findings, we speculate that following the repeated duplication of a Delta gene locus, an evolutionary alteration of the nervous system might be possible without alteration of the segmented body plan. A difference in the sensitivity of developmental processes to gene copy number may enable the dissociation of evolutionary trajectories of different organ systems. Whether this would be of advantage to the mutant individual would of course depend on local selective pressures.

Do differences in levels of Notch signalling components, for example, Notch receptors, underlie the observed differences between the responses of neurogenesis and segmentation to elevated Delta? From our data, it does not appear that signalling component levels are limiting, since there is no evidence of saturation in either embryonic context; both assays show changes between *Dover* and *Damascus* (Fig. 6). Rather it appears that the tissues show a difference in the gain of their outputs across the range of Delta input. These differences may be due to tissue-specific signal transduction components that confer distinct properties of information processing in lateral inhibition and synchronization mechanisms. Answering these questions will allow multicellular biological systems to be better understood in development and disease states, and also to be engineered with a greater range of spatial and temporal characteristics in the future.

## Methods

### Animals and embryos

Zebrafish (*Danio rerio*) adult stocks in the fish facilities of the Max Planck Institute of Molecular Cell Biology and Genetics and University College London were kept in 28 °C fresh water under a 14:10 h light:dark photoperiod. Embryos were collected within 30 min following fertilization and incubated in petri dishes with E3 media. Until the desired developmental stages^48^, embryos were incubated at 28.5 °C. For whole-mount *in situ* hybridization (ISH), PTU (1-phenyl 2-thiourea) at a final concentration of 0.003% was added before 12 h.p.f. to prevent melanogenesis. All WTs were *AB*, unless otherwise specified, and the *deltaD* mutant allele used was *aei*^*tr233* 16^ crossed onto the same *AB* background. Zebrafish experimentation was carried out in strict accordance with the ethics and regulations of the Saxonian Ministry of the Environment and Agriculture in Germany under licence Az. 74-9165.40-9-2001, and the Home Office in the United Kingdom under project licence PPL No. 70/7675.

### DAPT treatment and pulse-chase experiments

DAPT treatment was carried out as previously described^11^. In brief, 50 mM DAPT stock solution (Merck) was prepared in 100% DMSO (Sigma) and stored in a small volume at −20 °C. Embryos in their chorions were transferred to 12-well plates at 2 h.p.f. in 1.6 ml E3 medium with 20 embryos per well. A total of 50 μM DAPT in E3 medium was prepared immediately before the treatment. To prevent precipitation, the DAPT stock solution was added into E3 medium while vortexing, and then filtered by 0.22 μm PES syringe filter (Millipore). DAPT treatment was initiated at dome stage (4 h.p.f.) by replacing E3 medium with E3/DAPT medium. For pulse-chase experiments, DAPT was washed out at least twice with fresh E3 medium +0.03% PTU. Embryos were dechorionated and fixed at 36 h.p.f. All experiments were incubated at 28.5 °C, except for short operations, for example, washing out, which were at room temperature.

### Scoring segment defects and segment number

Embryos stained with the *cb1045* riboprobe marking mRNA from the *xirp2a* gene were visually scored under an Olympus SZX-12 stereomicroscope and images were acquired with a QImaging Micropublisher 5.0 RTV camera. Defective segmentation boundaries were scored following^11^ with modification. In short, any abnormal boundary was defined as defective, and two measures were collected for each left-right side of the embryo: an ALD, that is, the position of the first defective boundary and the FRS, that is, the anterior boundary position of the first non-defective segment after the DAPT wash-out at 9 ss. In all scoring, the first two somites and those posterior to the 30th were defined as non-defective since the first two were clutch-dependent and the posterior-most segments were too small for reliable analysis. Total segment number was counted in 36–39 h.p.f. embryos; embryos earlier than this stage had weaker *xirp2a* staining patterns. The re-synchronization rate is defined as the multiplicative inverse of the boundary difference between DAPT wash-out and FRS, that is, FRS minus 9.

### BAC recombineering

Red/ET recombination was carried out as published (Supplementary Fig. 1, upper panel)^49^,^50^,^51^. Briefly, the recombination was performed in an *E. coli HS996* strain carrying a toolbox plasmid, pRed-flp4. pRed-flp4 has a thermo-sensitive replication origin, *pSC101*, and contains anhydrotetracycline inducible *Flippase* (*Flp*) and an operon of homologous recombinases, *Redα*, *Redβ*, *Redγ* and *RecA*, controlled by a L-rhamnose-inducible promoter. The *venus*-*kanamycin* tagging cassette was generated by PCR from the plasmid template pR6K-venus in which the *R6K* origin provides background-free tagging because its replication cannot proceeded without a specific initiator^52^.

A commercially available BAC clone encompassing the *deltaD* locus (ID: CH1073-241D9) was first electroporated into *HS996*/pRed-flp4, then confirmed by colony PCR of overnight cultured bacterial colonies on LB-agar solid media with corresponding antibiotics. The positive clone of *HS996*/pRed-flp4/*deltaD*-BAC was cultured overnight in LB liquid media with antibiotics, and then 60 μl of the saturated culture was transferred to 1.8 ml fresh medium at 30 °C on Thermomixer shaker (Eppendorf) until its OD600 reached 0.2. Red expression was induced by adding 30 μl of 25% L-rhamnose to the liquid culture for 1 h at 37 °C. After the OD600 reached 0.4, 500 ng of the tagging cassette was eletroporated into *HS996*/pRed-flp4/*deltaD*-BAC, and then the culture was transferred into SOC medium without antibiotic for 1 h at 37 °C. After re-growth, the culture was centrifuged and transferred to LB medium or an LB-agar plate with 10 μg ml^−1^ kanamycin for overnight culture. The positive clones were screening by colony PCR for the region across the *deltaD* stop codon, and the kanamycin flanked by two FRT sites was then removed by the induction of Flp with 200 nM anhydrotetracycline in the medium.

To subclone, the vector backbone for recombination was amplified by PCR from a plasmid template, pNPC2, which is a p15A origin plasmid containing two I-SceI recognition sites, a *ClonNAT* resistant gene, and *ccdB* toxic gene for reducing recombination background. Homology arms targeting the 5′ upstream or the 3′ downstream *deltaD* locus resulting in a 14-Kbp fragment were designed to the 5′ ends of the subcloning primers. Recombination is similar to the tagging described above, with the exception that the final culture was plated on LB-Agar plates with 5 μg ml^−1^ ClonNAT (WERNER BioAgents). Positive clones were screened by colony PCR and plasmid DNA was prepared using a Qiagen Midi-Prep kit according to the manufacturer's instructions. The final construct, pNPC2-*deltaD*-*venus*, was verified by sequencing the tagging region and the conjunctions of the vector backbone to the subcloned fragment (MPI-CBG sequencing facility). The list of primer sequences and PCR conditions can be found as Supplementary Table 2.

### Microinjection and I-SceI-mediated transgenesis

Meganuclease transgenesis was performed as the previously described (Supplementary Fig. 1, lower panel)^23^. In brief, injection needles were pulled in preparation from 1.0 mm O.D. × 0.58 mm I.D capillaries (Harvard Apparatus) using a Flaming/Brown micropipette puller (Sutter Instruments), and meganuclease I-SceI (Roche) was stored in −80 °C as 2 μl aliquots.

Staged embryos from an incross of *aei*/*deltaD* homozygous adults were placed into a 1.5% agarose (Sigma) injection mould in 0.3 × Danieau's buffer and injected at the one cell stage. A 20 μl injection mixture was made immediately before injection from 14 μl of pNPC2-*deltaD*-*venus* (100 ng μl^−1^), 2 μl of 10 × I-SceI buffer, 2 μl of 50 mM MgCl_2_, and 2 μl I-SceI (10 U μl^−1^). A pneumatic Pico Pump PV 820 (World Precision Instruments) was used for injection. After loading the injection mixture into the needle with a microloader tip (Eppendorf), the injection volume was adjusted to ∼1 nl by measuring the diameter of bolus injected into a drop of mineral oil (Sigma) on the objective micrometre. The injection mixture was directly injected into the cytoplasm of the embryos. After injection, embryos were transferred into E3 medium and screened for positive Venus-YFP fluorescence around 24 h.p.f. I-SceI meganuclease-mediated transgenesis is known for its high transgenesis efficiency and resulting in variation of transgene copy number with few genome insertions^53^,^54^,^55^. The potential founders were screened by whole-mount ISH staining of transgene expression and the rescue of their *aei*^−/−^ background phenotype.

### Genomic DNA extraction and quantitative real-time PCR

Genomic DNA was extracted from either fin-clips of adults or twenty 5-days post fertilization embryos using a DNeasy Blood & Tissue Kit (Qiagen, Cat No./ID 69504) with RNaseA treatment as described in the manufacturer's instructions. The concentrations of genomic DNA extracts were then measured by Nanodrop 2000 (Thermo scientific) and adjusted to 6 ng μl^−1^. Quantitative real-time PCR was carried out using the Mx3000 Real-time PCR system (Stratagene) with its built-in acquisition/analysis software. To prepare the PCR, two replicates of each sample were loaded into 96-well plates with a final volume of 10 μl in each well, containing 5 μl 2 × SYBR Green master mix (ThermoFisher Scientific), 30 nM ROX free dye (ThermoFisher Scientific), 70 nM primer pair, and 3 μl genomic DNA. The PCR programme was 95 °C 15 min for initiation, 40 cycles of 95 °C 30 s, 60 °C 30 s and 72 °C 30 s, followed by the default dissociation analysis. Seven pairs of primers targeting the *deltaD* genome locus, *deltaC* genome locus or *venus* open reading frame were used (Supplementary Table 3), and the standard curve and PCR efficiency of each primer pair was determined by serial dilution of WT samples.

To analyze the samples, we first normalized the raw Ct. values from *deltaC* or *deltaD* primer pairs to the in-group WT values according to the PCR efficiency of each primer pair (Supplementary Table 3). The relative concentrations within each primer group were then divided by the averaged *deltaC* concentrations to calibrate the loading error. In the case of *venus* primer pairs, the raw Ct. values were normalized to one assigned transgenic sample. Thus, the copy numbers of each sample were averaged from the relative *deltaD* concentrations to WT samples and, if applicable, from the relative *venus* concentrations to each other. The copy numbers of *dover* and *damascus* were obtained from the average of at least three independent trials.

### Immunohistochemistry and quantification protein level

Dechorionated embryos were fixed in 4% paraformaldehyde/PBS for ISH. For whole-mount immunohistochemistry, 9 somite-stage embryos were washed several times with PBS/0.1% Tween and then permeabilized by washing with PBS/0.2% Triton X-100 for 30 min at room temperature (RT). Blocking solution (Roche) prepared in PBS/0.2% Triton X-100 was used to block at RT for 2 h, then the embryos were incubated with 1:100 PBS/0.2% Triton X-100 diluted mouse anti-DeltaD monoclonal antibody (Abcam) at 4 °C overnight. After primary antibody incubation, embryos were washed with PBS/0.2% Triton X-100 four times for 30 min, then incubated with 1:200 PBS/0.2% Triton X-100 diluted goat anti-mouse IgG conjugated with Alexa Fluor 488 (Invitrogen) for 2 h at RT. Secondary antibody was washed out with PBS/0.2% Triton X-100 at least four times for 30 min at RT. After staining, samples were gradually transferred into 87% glycerol/13% PBS and kept at 4 °C overnight before flat-mounting. ProLong Gold Antifade Reagent (Invirogen) was used as a mounting reagent.

To compare genotypes, embryos were stained in parallel in each experiment. Images from PSM of flat-mounted samples were acquired by a Zeiss LSM 510 META confocal laser-scanning microscope. Acquisition configurations were identical for all samples. Mean fluorescence intensity of the PSM was obtained from *z*-projection of three non-overlapping *z*-sections with 15 μm intervals, and the middle section was aligned to the centre of the notochord. The relative DeltaD levels in the posterior PSM were the average intensities obtained from circular areas of 68 μm diameter.

### Time-lapse imaging and analysis

Somitogenesis period was determined by multiple embryo time-lapse microscopy as previously described^56^,^57^. In brief, 20–25-staged embryos were dechorionated by forceps immediately before imaging. Dechorionated embryos were carefully aligned laterally on the mould made by 2% low-melting point agarose (Sigma-Aldrich) on the cell culture dish with glass bottom (CELLview). To prevent the embryos moving during imaging, buffered Tricaine (ethyl 3-aminobenzoate methanesulfonic acid) (Sigma-Aldrich) in a final concentration of 0.016% in E3 medium was used for anaesthesia. Time-lapse images were acquired by an inverted Zeiss Axiovert 200M microscope with an Andor iXOn 888 EM-CCD camera and software controlled motor stage. Temperature during imaging was controlled by a Warner Thermal Cooling Module TCM-1 (Warner Instruments), and recorded during imaging by an electro-thermometer Voltcraft Plus K202. Imaging was started at bud stage (10 h.p.f.) and lasted for 16.5 h; the acquisition and image post-processing were performed using Andor IQ2.0 software. To treat with DAPT, embryos at 4 h.p.f. were transferred to 50 μM DAPT or DMSO control in E3 medium. Four-chamber glass bottom dishes holding WT or transgenic embryos with or without DAPT were used for imaging.

To calculate the somitogenesis period, the timing of somitic furrow emergence in the trunk was assessed in each embryo from the time-lapse movies for segments 4–19. The slope of linear regression from time point versus somite number was calculated using Microsoft Excel. Somitogenesis period is the slope multiplied by time interval of the time-lapse movie. The somitogenesis period of each genotype was obtained from at least three independent trials at the same temperature setting.

The measurement of the axis elongation and PSM shortening was performed as previously published^2^,^18^,^39^. Briefly, in the time-lapse movie collected above, a measurement was made at each relevant time interval using the segmented line tool in Fiji. This line extended from the posterior tip of the tailbud to either the anterior end of the head or the most recently formed somite boundary, respectively, and followed the course of the notochord. The time 0 was defined as 5 somite-stage in the present study.

### Measurement of anterior wavelength from *her1* whole-mount ISH

A total of 4 to 20 somite-stage embryos were stained with the *her1* riboprobe according to published ISH protocol^58^ and imaged laterally on an Olympus SZX-10 stereomicroscope equipped with a QImaging Micropublisher 5.0 RTV camera. TIFF-formatted images were converted to inverted grayscale images from the red channel to give higher signal/noise ratio than the original RGB images.

To measure the intensity distribution along the axis, segment lines with 35 pixels (36.6 μm) width were drawn from anterior to the most anterior *her1* stripe to the end of PSM (Fig. 3a, top-left panel). The anterior wavelengths were calculated from the intervals between the first two peaks (Fig. 3a). The anterior border of the first peak, *X*_0_, is defined by the intersection of the background line with the linear regression line with the highest *r*^2^ value starting from the inflection point of the anterior side of the peak, extending anteriorly along the curve of the peak. *her1* expression length, *her1*-L, is the length in pixels from the *X*_0_ to the end of line measurement.

### Measurement of cell densities/areas in *islet1* whole-mount ISH

Four somite-stage embryos were hybridized with the *islet1* riboprobe and imaged as described above. TIFF-formatted images were adjusted by auto contrast in Adobe Photoshop CS4 and converted to grayscale images from the red channel.

To measure the cell density of PMN and RB, we used a plug-in, Peak Finder (Johannes Schindelin, unpublished) in Fiji^59^. Along the PMN or RB in the anterior axis (Fig. 4a, lower right panel), we drew a line 5 pixels (3.78 μm) wide and 228.1±40.2 μm (PMN) or 233.9±40.4 μm (RB) long, then analyzed the number of peaks in pixel intensity along this line by Peak Finder. To measure TG areas, monochrome bitmap images were generated by watershedding images using Adobe Photoshop CS4 with various levels to match the black areas resembling the TG areas. TG areas were thus obtained from the number of black pixels.

### Calculation of segmentation period from anterior wavelength

In *Damascus*, the anterior wavelength is shorter than in WT, which we expect to affect the frequency contribution from the Doppler effect (Supplementary Note 1). Here, we tested how this altered Doppler contribution relates to the observed difference in the segmentation rate between WT and *Damascus*. As a reference for the relative contribution of the Doppler effect to the total rate of segmentation, we used the measurements on the *Looping* live reporter^2^. From this we estimated that the Doppler effect accounts for 22% of the observed rate of segmentation (Supplementary Note 2). The rate of segmentation in WT at 28.5 °C is 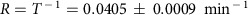, where *T* is the segmentation period, which yielded an expected Doppler contribution of *D*=22% · *R*=0.0089±0.0002, min^−1^. Using the fact that the tissue shortening speed is the same in WT and *Damascus* and taking into account the effects of the altered wavelength on the Doppler contribution, we expect in *Damascus* a Doppler contribution of


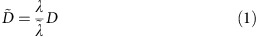


where *λ* is the anterior wavelength in WT and 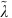 is the anterior wavelength in *Damascus* (Supplementary Text 1). For the case that the altered Doppler contribution is the only change in the wave pattern affecting the segmentation rate, we obtained an estimate for the segmentation rate in Damascus as 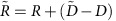 and the segmentation period follows as 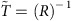. The measured relative wavelength 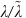, the inferred Doppler contribution 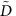 and resulting segmentation period 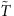 at each of the time-points measured in Fig. 3b is given in Supplementary Table 1.

### Illustration of the effects of coupling on the wave pattern

We used a theory of coupled oscillators that takes coupling delays into account to demonstrate how the wavelength of the resulting wave pattern depends on coupling (Supplementary Note 3). We described the wave pattern in the PSM by a one-dimensional phase field along the elongating body axis in a reference frame co-moving with the tailbud tip (Supplementary Fig. 4a). In this reference frame, cells move in an anterior direction to become part of the formed segments, represented by the arrested wave pattern. Wave patterns emerge from a position-dependent frequency profile along the body axis with maximum frequency at the tailbud tip and oscillations gradually slow down as cells approach the anterior end (Supplementary Fig. 4b). For a constant PSM length, this system attains a state with a time-periodic wave pattern. The time-independent phase profile describes the spatial distribution of the waves along the PSM (Supplementary Fig. 4c–f). The wave pattern can be characterized by its local wavelength and the total number of waves in the PSM. Here we focused on the wavelength at the anterior end, which is experimentally evaluated in this study (Fig. 3b; Supplementary Fig. 2). Not all solutions for wave patterns were robust under small perturbations of the phase field. In certain parameter regions, small perturbations grow exponentially and prevent the system from forming a stable wave pattern. We performed a linear stability analysis that reveals the regions in which the pattern is stable (Supplementary Note 3). In certain parameter regions, the system exhibited multiple solutions for a given set of parameters. However, here we only considered parameter regions that enable a single stable solution, which corresponds to a robust pattern forming system (Supplementary Fig. 5A). In the regions with stable wave patterns, we simulated the system numerically and characterized the resulting wave patterns by their wavelength and the number of waves (Supplementary Fig. 5). Alterations in the strength and delay of coupling led to a variety of different wavelengths and number of waves in the PSM (Supplementary Figs 4C–F and 5C,D).

### Statistics

Values were presented as mean±s.d. unless otherwise described. Student's *t*-test with the parameters of two-tailed and unequal variance was performed in Microsoft Excel.

### Data availability

The data that support the findings of this study are available from the corresponding author upon request.

## Additional information

**How to cite this article:** Liao, B.-K. *et al*. Faster embryonic segmentation through elevated Delta-Notch signalling. *Nat. Commun.* 7:11861 doi: 10.1038/ncomms11861 (2016).

## Supplementary Material

Supplementary Information Supplementary Figures 1-5, Supplementary Tables 1-3, Supplementary Notes 1-3, Supplementary References
