# Supplementary material for: Faster embryonic segmentation through elevated Delta-Notch signalling
Source: Nat Commun. 2016 Jun 15;7:11861. doi: 10.1038/ncomms11861 (PMC4912627; doi:10.1038/ncomms11861)
Supplement: Supplementary Information — Supplementary Figures 1-5, Supplementary Tables 1-3, Supplementary Notes 1-3, Supplementary References [file ncomms11861-s1.pdf]

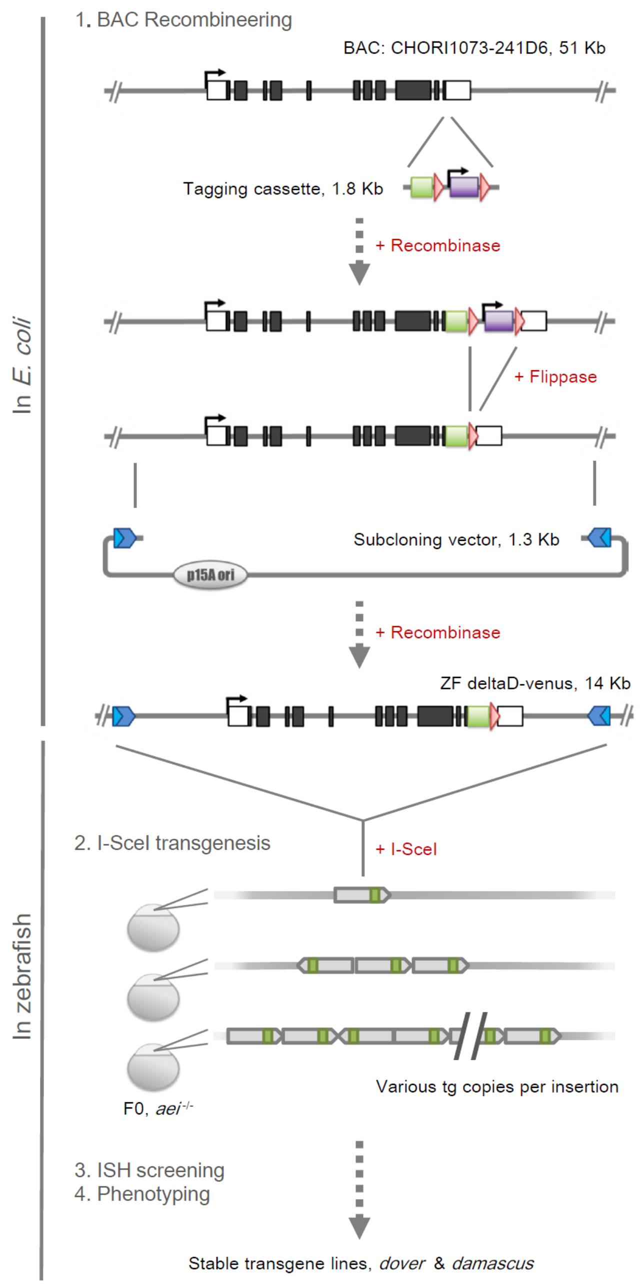

Supplementary Figure 1. Schematic representation of BAC recombineering and meganuclease transgenesis pipeline

Flowchart for generating transgenic lines with DeltaD copy-number variation. A commercially available BAC clone (clone ID: CH1073-241D9) containing the zebrafish *deltaD* genomic locus was first tagged with *venus-yfp* and then subcloned into a p15A ori vector within the region flanked by 2 I-SceI meganuclease sites. The subcloned 14 Kb *deltaD-venus* construct including approximately 5 Kb of 5' upstream genomic DNA was microinjected into zebrafish *aei*$^{-/-}$ embryos using an I-SceI meganuclease-mediated approach. Potential founders carrying various transgene copies in single insertion loci were screened by whole-mount ISH for the presence of *yfp* mRNA and morphologically rescued somite boundaries. Blue pentagon, I-SceI site; green box, *venus* coding region; red triangle, FRT site; purple box, kanamycin-resistance gene.

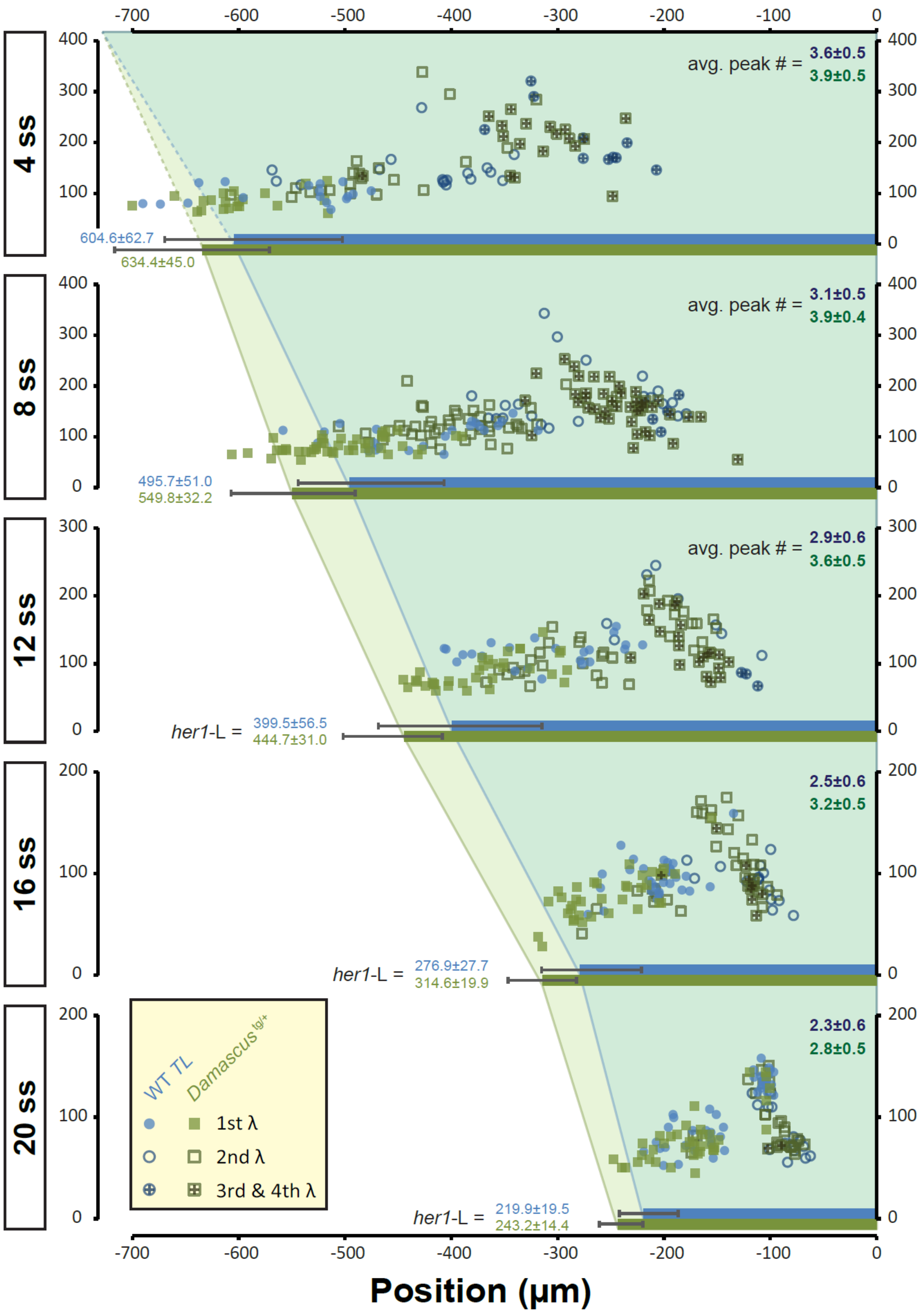

Supplementary Figure 2. Phase profiles of *her1* expression pattern revealed by wavelength versus position in the PSM

The position of wavelength was registered to the posterior end of PSM as 0 and negative value is toward the anterior. The bar charts at the bottom of each panel are mean *her1*-L with 5th and 95th percentiles as the error bars, and the corresponding values (mean ± s.d.) are indicated in front of the bar charts. 1st λ, distance between 1st and 2nd *her1* peaks; 2nd λ, distance between 2nd and 3rd *her1* peaks; 3rd and 4th λ, etc. Mean number of gene expression peaks (avg. peak #, values in mean ± s.d.) per embryo for the two conditions are given in the upper right corner of each panel.

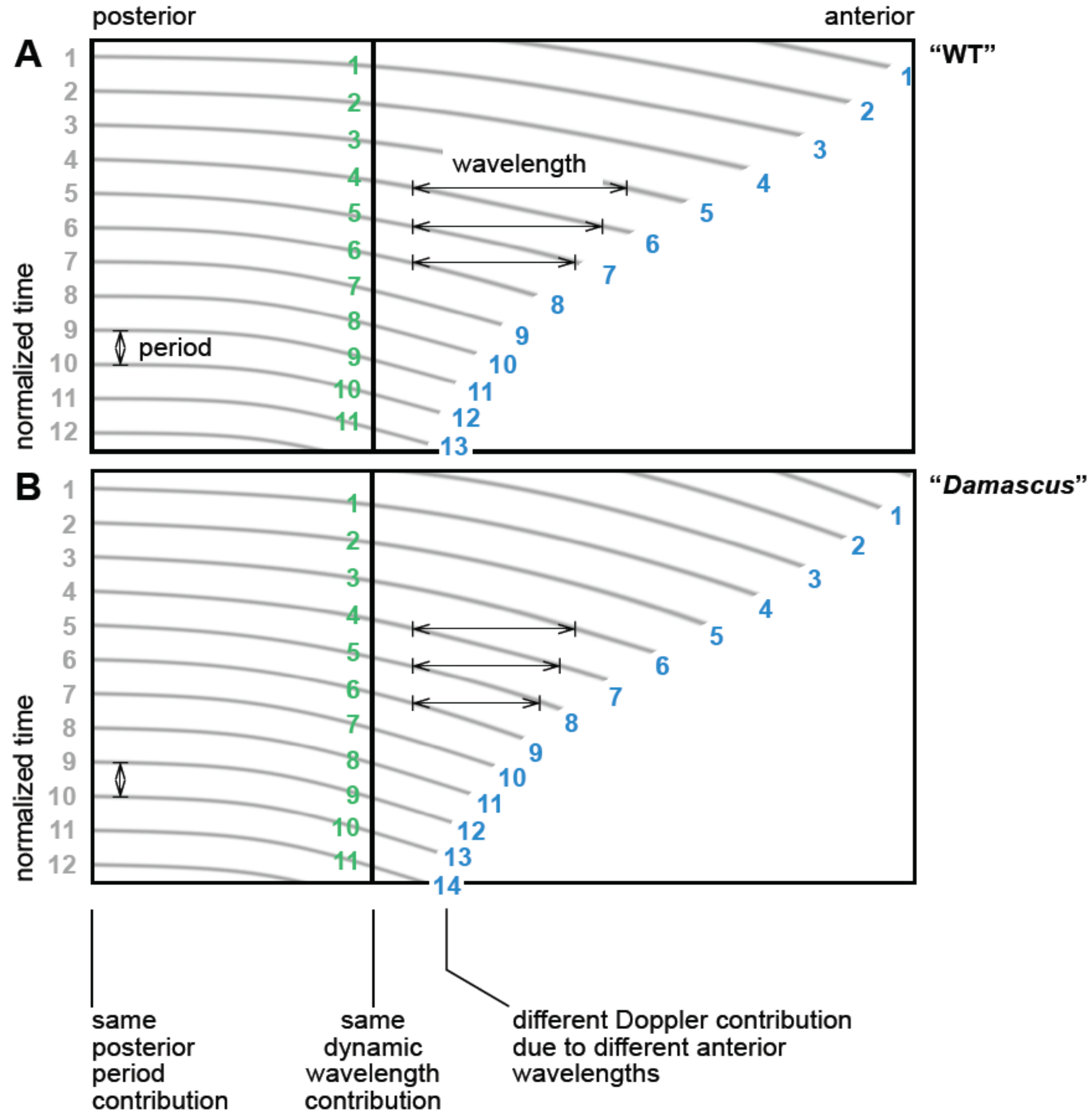

Supplementary Figure 3. An increased Doppler contribution due to a changed wave pattern can increase the segmentation rate

(A) Kymograph representation of waves moving across the wildtype PSM (WT). Gray lines indicate the motion of the wave peaks. Due to the dynamic change of the wavelength in the vicinity of the anterior end, a smaller number of waves arrives at a fixed position near the anterior end (green numbers) than is emitted at the posterior time during the same time interval (gray numbers). Due to the Doppler effect caused by the anterior end moving into the waves, a larger number of waves arrives at the anterior end (blue numbers). **(B)** Compared to A, the wavelength in the anterior is shorter giving rise to a stronger Doppler effect, leading to a larger number of waves arriving at the anterior end (blue numbers). For simplicity, we here show a scenario where all other contributions remain unchanged (gray and green numbers).

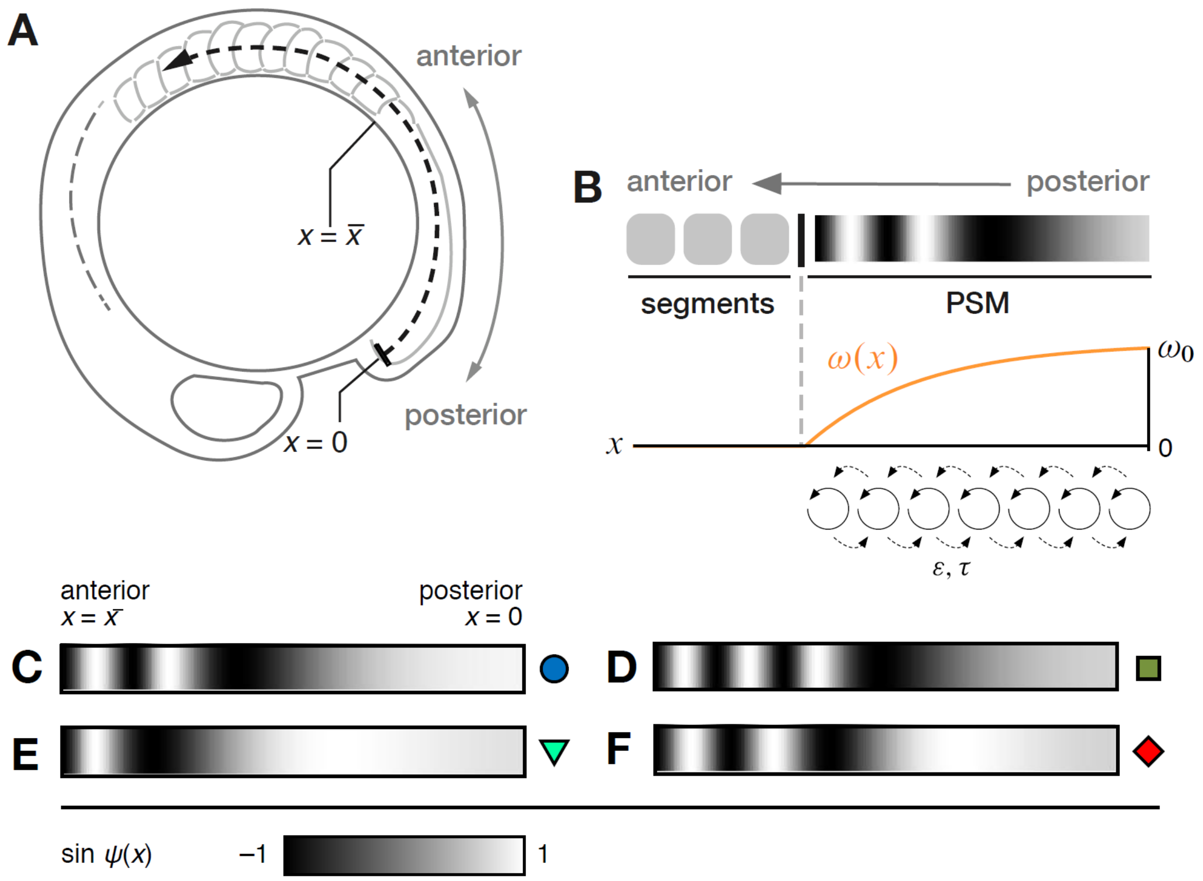

Supplementary Figure 4. Time-periodic wave patterns in the "segmentation clock" with altered coupling strength and coupling time delays

(A) Schematic depiction of the zebrafish embryo. The dashed black curve shows the embryonic body axis, $x = 0$ marks the posterior tip, $x = \tilde{x}$ marks the anterior end of the PSM. **(B)** Shape of the frequency profile $\omega(x)$. Panels A and B adapted from [1]. Oscillators are coupled with coupling strength ε and coupling delay τ. (C–F) Examples of time-periodic wave patterns for different coupling strengths and coupling delays: **(C)** $\varepsilon = 0.07$ min^{-1}, $\tau = 20.75$ min; **(D)** $\varepsilon = 0.09$ min^{-1}, $\tau = 17$ min; **(E)** $\varepsilon = 0.07$ min^{-1}, $\tau = 26$ min; **(F)** $\varepsilon = 0.04$ min^{-1}, $\tau = 20.75$ min. The other parameters are $\omega_0 = 0.2205$ min^{-1}, $\mu = 0.249$ cd min^{-1}, $\tilde{x} = 39$ cd, and $\sigma = 27$ cd, where 'cd' denotes cell diameters [2]. The colored symbols to the right of the pattern indicate the location of the parameter set in Supplementary Fig. 5D. See Supplementary Note 1 for details.

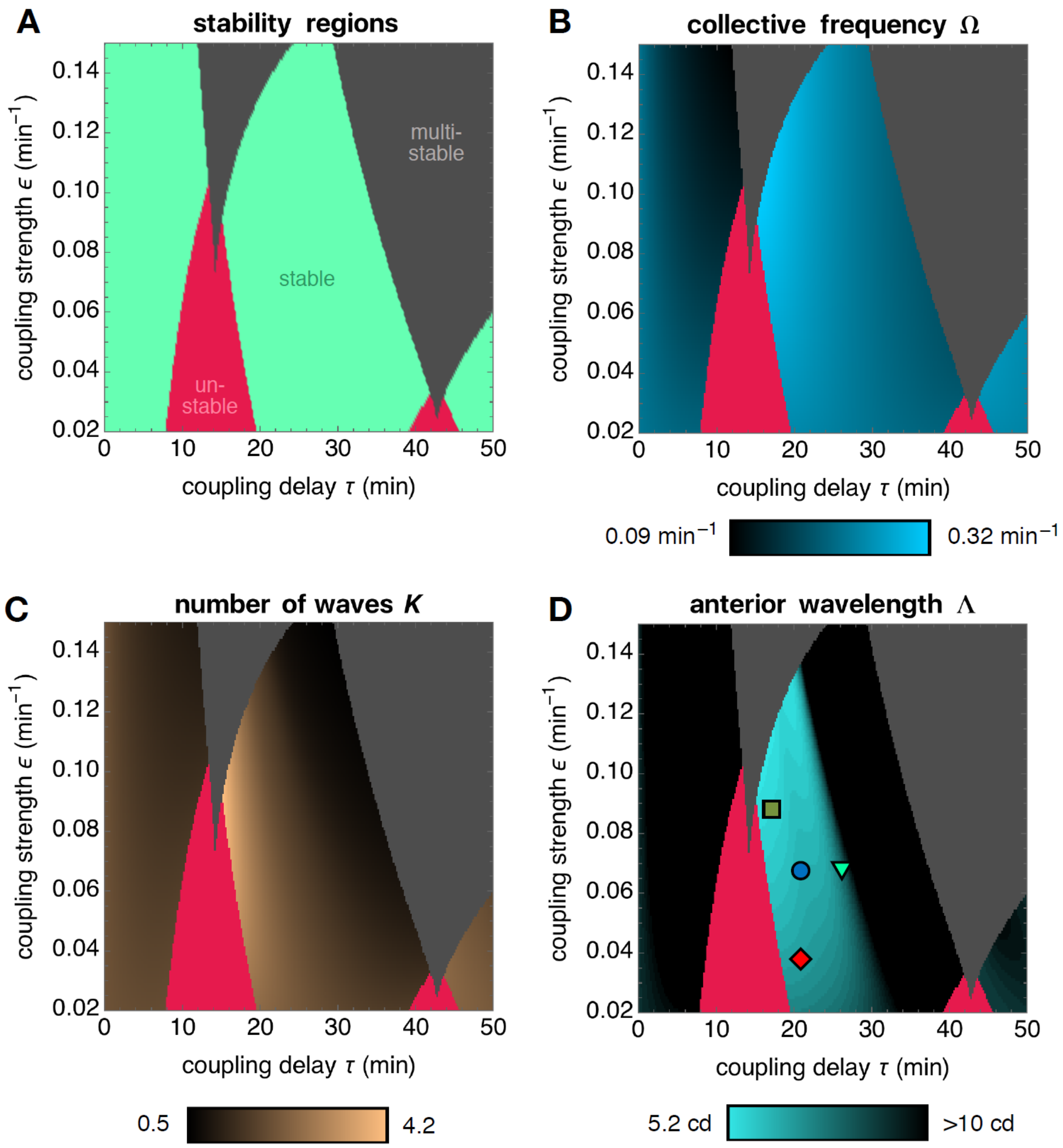

Supplementary Figure 5. Stability, frequency and wavelength of time-periodic wave patterns

Properties of time-periodic wave patterns as a function of the coupling delay τ and the coupling strength ε. **(A)** Regions where the wave pattern is stable (green), unstable (red) and where more than one solution for the wave pattern exists (dark gray). **(B)** Collective frequency Ω (blue), determined by Eq. (S14) in Supplementary Note 1, in the regions with a single stable solution. Other regions as in A. **(C)** Number of waves *K* (brown), Eq. (S17), in the regions with a single stable solution. Other regions as in A. **(D)** The wavelength of the pattern at the anterior end (cyan), Eq. (S16). Other regions as in A. Dots indicate parameter values for which the corresponding wave pattern is shown in Supplementary Fig. 4. The blue dot marks the wildtype parameters from [2] (ε = 0.07 min^{-1}, τ = 20.75 min) and the green square marks a parameter set that yields a shorter anterior wavelength as observed in *Damascus* (ε = 0.09 min^{-1}, τ = 17 min). Green triangle ε = 0.07 min^{-1}, τ = 26 min; red diamond ε = 0.04 min^{-1}, τ = 20.75 min. The other parameters are ω_0 = 0.2205 min^{-1}, μ = 0.249 cd min^{-1}, $\tilde{x}$ = 39 cd, and σ = 27 cd, where 'cd' denotes cell diameters [26]. See Supplementary Note 1 for details.

Supplementary Table 1. Inferred segmentation periods from Doppler effect

Somite Stage	Relative Wavelength $(\lambda/\tilde{\lambda})$	Inferred Doppler contribution	Inferred Period (min)
4	1.15 ± 0.07	0.0103 ± 0.0007	23.9 ± 0.7
8	1.28 ± 0.07	0.0114 ± 0.0007	23.2 ± 0.6
12	1.33 ± 0.07	0.0118 ± 0.0007	23.0 ± 0.6
16	1.22 ± 0.08	0.0109 ± 0.0007	23.5 ± 0.7
20	1.32 ± 0.09	0.0118 ± 0.0009	23.0 ± 0.7
Average	1.26 ± 0.03	0.0112 ± 0.0003	23.3 ± 0.3

Relative wavelength, inferred Doppler contribution and inferred Period is mean ± s.e.m., where the errors are obtained by Gaussian error propagation using the error of the wavelength ratio and the error of the wildtype period. The segmentation period measured by timelapse microscopy in wildtype and *Damascus* was 24.7 ± 0.02 min and 23.1 ± 0.07, mean ± s.e.m. (Fig. 2D).

Supplementary Table 2. Primers for recombineering

Primer name	Primer sequence (5'-3')	PCR conditions
Tagging Fwd	ATTTAGATATCTGAATGCTAAACTAACGTTTTGTTTCCTCGTTTCAGGTGAG CTCAGGAGGTAGCGG	98ºC – 5' (initial),<br>98ºC – 10''<br>68ºC – 15''<br>72ºC – 30'' (30x),<br>72ºC – 10' (final)
Tagging Rev	TGGAGTAAAACCTCCTGTAGAACAAGATGAGCTACAGCTCTGTTGTGGCGG GCAGATCGTCAGTCAG	
Subcloning Fwd	ATGTAAAAATGGCTTGCCGTATTTTTTTGAAAAATACAATAATAAGTGTGT GTGTTGCAACGAACAGG	98ºC – 5' (initial),<br>98ºC – 10''<br>68ºC – 15''<br>72ºC – 30'' (30x),<br>72ºC – 10' (final)
Subcloning Rev	AGAACGACGCGCCGGAACAGGAAGTAAACGCAACATTGTAAACACCAGCG CGCCAACCCAGCTTTCT	
Colony PCR Fwd	AAGTCCAAAACCTTTCCTTT	98ºC – 5' (initial),<br>98ºC – 10''<br>52ºC – 10''<br>72ºC – 20'' (40x),<br>72ºC – 10' (final)
Colony PCR Rev	TCTGGTTTGAGCAGCATTAC	
Sequencing 1	GCCTGGACACTGCCCTGTAT	Tm = 62ºC
Sequencing 2	GATTACGCGCAGACCAAAAC	Tm = 60ºC
Sequencing 3	= Subcloning Fwd	Tm = 52ºC
Sequencing 4	= Subcloning Rev	Tm = 55ºC

All primers were synthesized by biomers.net. For the primers longer than 50 bp, HPLC purification was chose. Underlined, homology arm.

Supplementary Table 3. Primers for real-time PCR

Primer name	Primer sequence (5'-3')	Size (bp)	PCR efficiency
deltaD Q1 Fwd	ACCGACTCCACTGATGACCT	169	1.998
deltaD Q1 Rev	CCTCGCCTACTGTTAGATGC		
deltaD Q2 Fwd	TAAGGAGTCAGGATGTGGGTAT	198	1.902
deltaD Q2 Rev	GATGTGAATGGCTTGAGGTG		
deltaC Q2 Fwd	CACATAATACAGGGCGGAACA	190	2.017
deltaC Q2 Rev	TTGGAAACCCACGCTAACC		
deltaC Q3 Fwd	TCTCCCTATCCTTTCTAAACAGC	54	1.920
deltaC Q3 Rev	GTTCTCCTCACTACAGCCAGAC		
venus Q2 Fwd	GTCCAGGAGCGCACCATCTT	205	2.019
venus Q2 Rev	TGCCGTTCTTCTGCTTGTCG		
venus Q4 Fwd	GAGGGCGATGCCACCTACGG	275	1.804
venus Q4 Rev	AGCTCGATGCGGTTCACCAG		
venus Q6 Fwd	AAACGGCCACAAGTTCAGCG	128	1.869
venus Q6 Rev	CCAGGGTGGTCACGAGGGTG		

PCR efficiency is the fold change of product concentration from one PCR cycle to next PCR cycle. All primers were synthesized by biomers.net.

Supplementary Note 1:
Changes in the wavelength can alter the segmentation rate

Here we illustrate how a change in the wavelength can increase the segmentation rate by enhancing the Doppler effect that occurs due to PSM shortening over time.[3] The wave pattern is described by a one-dimensional phase field $\phi(x,t)$, which indicates the local state of oscillation at time t and position x as measured from the posterior tip along the body axis, see Fig. S4A.[1] The position of the anterior end is given by $x = \bar{x}(t)$. As the PSM shortens over time, $\bar{x}$ continously decreases over time. The phase at the anterior end is given by $\phi_\mathrm{A}(t) = \phi(\bar{x}(t), t)$ and the segmentation rate R, which is proportional to the anterior frequency, can be expressed as[1,3]

$$R = \frac{1}{2\pi}\frac{\mathrm{d}\phi_\mathrm{A}}{\mathrm{d}t} = P + W + D \,, \tag{1}$$

where the frequency P at the posterior tip $x = 0$, the dynamic wavelength contribution W and the Doppler contribution D are given by

$$P = \frac{1}{2\pi}\frac{\partial\phi}{\partial t}\bigg|_{x=0} \,, \qquad W = \frac{1}{2\pi}\frac{\partial\phi}{\partial t}\bigg|_{x=\bar{x}} - P \,, \qquad D = \frac{u}{\lambda} \,. \tag{2}$$

Here, $u = \mathrm{d}\bar{x}/\mathrm{d}t$ is the shortening speed of the PSM and λ is the wavelength of the pattern at the anterior end, given by

$$\lambda = \frac{2\pi}{\partial\phi/\partial x}\bigg|_{x=\bar{x}} \,. \tag{3}$$

We now assess how the segmentation rate R is affected if the wavelength of the wave pattern is altered. If ϕ describes a reference wave pattern (e.g., wildtype), then a correspondingly altered wave pattern (e.g., *Damascus*) can be expressed as

$$\tilde{\phi}(x,t) = \phi(x,t) + \theta(x,t) \,, \tag{4}$$

where θ describes the differences in the pattern that may depend on time. To illustrate the effects of a changed wave pattern in a simple example, we here consider a time-independent change of the wavelength, $\theta = \theta(x)$. In this case, since $\partial\tilde{\phi}/\partial t = \partial\phi/\partial t$, both the posterior frequency contribution P as well as the dynamic wavelength contribution W are unaffected by such a change in the wave pattern. However, the corresponding wavelength at the anterior end is changed as

$$\tilde{\lambda} = \frac{2\pi}{\partial\tilde{\phi}/\partial x}\bigg|_{x=\bar{x}(t)} = \frac{2\pi}{\partial\phi/\partial x + \mathrm{d}\theta/\mathrm{d}x}\bigg|_{x=\bar{x}(t)} \,. \tag{5}$$

Given that the shortening speed u of the PSM is the same, Eq. (2) implies that the Doppler contribution $\tilde{D} = u/\tilde{\lambda}$ for the changed wave pattern can be expressed in terms of the Doppler contribution D of the reference wave pattern as

$$\tilde{D} = \frac{\lambda}{\tilde{\lambda}} D \,. \tag{6}$$

Using Eqs. (3) and (5), the ratio of wavelengths can explicitly be given as

$$\frac{\lambda}{\tilde{\lambda}} = 1 + \frac{(\mathrm{d}\theta/\mathrm{d}x)}{(\partial\phi/\partial x)}\bigg|_{x=\bar{x}} \,. \tag{7}$$

Moreover, the segmentation rate $\tilde{R}$ resulting from the change in the pattern is given by

$$\tilde{R} = P + W + \tilde{D} = R + (\tilde{D} - D) \,. \tag{8}$$

Supplementary Note 2:
Estimating the relative magnitude of the Doppler effect

In Ref. 3, quantified wave patterns from the transgenic live reporter zebrafish line 'Looping' have been used to generate an average experimental phase map $\phi(x,t)$ from 18 embryos. We use this phase map to determine the average contribution of the Doppler effect to the oscillation frequency at the anterior end,

$$q = \frac{1}{T}\int_0^T \frac{D(t)}{R(t)}\,\mathrm{d}t\ . \tag{9}$$

Here, D and R are the Doppler contribution and the anterior frequency (which coincides with the segmentation rate) as defined in Eqs. (1) and (2), and $T = 500\,\mathrm{min}$ is the duration over which the average phase map has been determined. Using the functional form for $\phi(x,t)$ and the parameters provided in Ref. 3, we obtain $q \simeq 0.22$.

Supplementary Note 3:
Effects of coupling strength and coupling delays on the wave pattern

In the previous sections, we have shown that an altered wave pattern can lead to a different segmentation rate but we have not addressed how such an alteration of the wave pattern can arise in the first place. Here we illustrate how changes in the strength and the delay of intercellular communication can give rise to changes in the wave pattern. For simplicity, we consider a scenario in which the PSM has a constant length throughout segmentation. Even though this is not the case *in vivo*, where the PSM continuously changes its length during segmentation, this scenario enables illustration of the effects of coupling on the overall shape of the wave pattern in a simple setting.

Continuum theory of coupled oscillators with coupling delays

To describe the dynamics of the phase field $\phi(x,t)$ introduced in the previous section, we use a continuum theory of coupled phase oscillators that takes into account delays in intercellular communication.[2] The phase dynamics is given by

$$\frac{\partial\phi}{\partial t} = \omega + \varepsilon\sin(\phi_\tau - \phi) - \frac{\varepsilon\xi^2}{2}\sin(\phi_\tau - \phi)\left(\frac{\partial\phi_\tau}{\partial x}\right)^2 + \frac{\varepsilon\xi^2}{2}\cos(\phi_\tau - \phi)\frac{\partial^2\phi_\tau}{\partial x^2}\ , \tag{10}$$

where $\phi_\tau(x,t) = \phi(x,t-\tau)$ is the time-delayed phase variable with τ being the coupling delay, $\omega(x,t)$ is a position-dependent frequency profile, ε is the coupling strength, and ξ is the 'lattice spacing', which corresponds to the typical cell diameter. In our simplified model, body axis elongation proceeds with a constant velocity v. We transition into a comoving reference frame, in which $x = 0$ marks the posterior tip of the PSM and the x-axis points in anterior direction, see Fig. S4A. The position $x = \bar{x}$ marks the position of the anterior end of the PSM. In this reference frame, the phase field is given by $\varphi(x,t) = \phi(x - vt, t)$. The dynamics for φ is given by

$$\left(\frac{\partial}{\partial t} + v\frac{\partial}{\partial x}\right)\varphi(x,t) = \omega(x) + \varepsilon\sin\big(\varphi(x - v\tau, t-\tau) - \varphi(x,t)\big) + \mathcal{O}(\xi^2)\ , \tag{11}$$

where we have neglected higher order corrections in the lattice spacing ξ. Following Refs. 2 and 4, we employ a frequency profile of the form

$$\omega(x) = \begin{cases} \omega_0 \dfrac{1 - \mathrm{e}^{(x-\bar{x})/\sigma}}{1 - \mathrm{e}^{-\bar{x}/\sigma}} & 0 \le x \le \bar{x} \\ 0 & x > \bar{x} \end{cases} , \tag{12}$$

where $\bar{x}$ is the length of the PSM and σ is a length scale that determines the shape of the frequency profile, see Fig. S4B. Such a frequency profile describes a gradual slowdown of oscillations across the PSM in anterior direction, which leads to the generation of waves in anterior direction.[1,2,4–6]

Time-periodic wave patterns

For a constant PSM length, this system attains a state with a time-periodic wave pattern,[2]

$$\varphi(x,t) = \Omega t + \psi(x) , \tag{13}$$

whose recurrence period $T = 2\pi/\Omega$ coincides with the period of segmentation. The time-independent phase profile ψ describes the spatial distribution of the waves along the PSM. The collective frequency of the pattern is determined by the equation[2]

$$\Omega = \omega_0 - \varepsilon \sin \Omega\tau . \tag{14}$$

This implicit equation does not possess a closed solution in Ω. Depending on the value of τ, Eq. (14) can have multiple solutions corresponding to different collective states with different frequencies. The phase profile ψ is determined by the equation

$$\Omega + v\frac{\mathrm{d}\psi}{\mathrm{d}x} = \omega(x) + \varepsilon \sin\big(\psi(x - v\tau) - \psi(x) - \Omega\tau\big) \tag{15}$$

with boundary condition $\psi(x)|_{-v\tau<x<0} = 0$.[2] From Eq. (15), it can be seen that both the coupling strength ε as well as the coupling delay τ determine the shape of the phase profile and thus the wave pattern.

The wave pattern can be characterized by its local wavelength and the total number of waves in the PSM. Here we focus on the wavelength at the anterior end $x = \bar{x}$, which is experimentally evaluated in this study, see Fig. 3B in the main text and Fig. S2. The total spatial extension Λ of the anteriormost wave in the direction of wave propagation is determined by the relation

$$\psi(\bar{x}) - \psi(\bar{x} - \Lambda) = 2\pi . \tag{16}$$

If the wavelength λ, defined by Eq. (3), does not vary strongly over the length Λ, both quantities coincide as can be seen from expanding Eq. (16) to first order in Λ.

The number K of waves that simultaneously travel across the PSM is given by

$$K = \frac{\psi(0) - \psi(\bar{x})}{2\pi} . \tag{17}$$

Stability of the pattern

Not all solutions for wave patterns are robust under small perturbations of the phase field. For certain configurations of the phase field, small perturbations exponentially grow and drive the system into a

different dynamical state. Such unstable wave patterns can immediately be ruled out as biologically irrelevant.

A full stability analysis for general solutions of the nonlinear delay equation (11) is a formidable task. However, for a given set of parameters and a specific solution Ω for the collective frequency, an indication of whether the corresponding pattern is stable or not can be obtained by considering the case of a flat phase profile, $\psi(x) = 0$, which is a solution for a flat frequency profile $\omega(x) = \omega_0$. In this case, linear stability analysis of Eq. (15) reveals that the solution is stable if and only if $\gamma > 0$, where

$$\gamma = \varepsilon \cos \Omega\tau \; . \tag{18}$$

This condition is derived in the paragraph below. The frequency profile (12) exhibits a roughly flat region in the vicinity of the posterior tip $x = 0$ (the tailbud region) for which the thus derived stability criterion approximately holds.

Linear stability analysis. We perform a standard linear stability analysis of the system defined by Eq. (11) for the homogeneous state $\varphi(x,t) = \Omega t$. For the phase field, we make the ansatz $\varphi(x,t) = \Omega t + \rho(x,t)$, where ρ is a small perturbation. Substituting this ansatz in Eq. (11) and expanding to first order in ρ yields the governing equation for the perturbation,

$$\frac{\partial \rho}{\partial t}(x,t) + v\frac{\partial \rho}{\partial x}(x,t) = \gamma\big[\rho(x - v\tau, t-\tau) - \rho(x,t)\big] + \mathcal{O}(\rho^2) \; , \tag{19}$$

where γ has been defined in Eq. (18). To proceed, we express the perturbation in terms of its spatial Fourier transform,

$$\rho(x,t) = \int \frac{\mathrm{d}k}{2\pi} \mathrm{e}^{-\mathrm{i}kx} \hat{\rho}(k,t) \; . \tag{20}$$

Using this relation in Eq. (19) and dropping all terms of order ρ^2 and higher yields

$$\frac{\partial \hat{\rho}}{\partial t}(k,t) - \mathrm{i}kv\hat{\rho}(k,t) = \gamma\big[\mathrm{e}^{\mathrm{i}kv\tau}\hat{\rho}(k,t-\tau) - \hat{\rho}(k,t)\big] \; . \tag{21}$$

Note that this equation now only contains derivatives and delayed arguments in t. To obtain the characteristic equation, we make the exponential ansatz $\hat{\rho}(k,t) = \mathrm{e}^{\lambda_k t}$, where the perturbation response rate λ_k depends on the wavenumber k. This yields

$$\lambda_k - \mathrm{i}kv = \gamma \mathrm{e}^{\mathrm{i}kv\tau}\mathrm{e}^{-\lambda_k\tau} - \gamma \; . \tag{22}$$

The homogeneous solution $\varphi(x,t) = \Omega t$ is stable if all solutions λ_k to Eq. (22) satisfy $\operatorname{Re}\lambda_k < 0$ for all k. Defining $\bar{\lambda}_k = \lambda_k - \mathrm{i}kv$, we can express Eq. (22) in the simpler form

$$\gamma = \mathrm{e}^{\bar{\lambda}_k\tau}(\bar{\lambda}_k + \gamma) \; . \tag{23}$$

Note that $\operatorname{Re}\bar{\lambda}_k = \operatorname{Re}\lambda_k$. For equations of the type Eq. (23) it has been shown that $\operatorname{Re}\bar{\lambda}_k < 0$ if and only if $\gamma > 0$.[7]

Numerical results

Fig. S5A shows the stability of the time-perodic state as a function of the coupling delay τ and the coupling strength ε, indicating unstable regions in red ($\gamma < 0$), stable regions in green ($\gamma > 0$), and multistable regions in dark gray (multiple solutions of Eq. (14) in Ω). The chosen set of parameters

has been used earlier to describe segmentation in zebrafish.[2] We consider as biologically relevant only such parameter regions that enable a single stable solution (green regions in Fig. S5A), which corresponds to a robust pattern forming system.[8] Fig. S5B shows the collective frequency Ω of the time-perodic state, determined by Eq. (14), in regions where there exists a unique solution. Both the coupling strength and the coupling delay regulate the collective frequency. Fig. S5C shows the number of waves K of the wave pattern in the PSM, Eq. (17). Fig. S5D shows the length Λ of the anteriormost wave of the pattern at the anterior end of the PSM, Eq. (16). The anterior wavelength exhibits a non-monotonic depedence on both the coupling strength and the coupling delay. Fig. S5D illustrates the wave patterns for selected points in parameter space. Differences in the wavelength and the number of waves are clearly visible. The blue dot marks the wildtype parameters from Ref. 2 (see also Fig. S4C). The green square marks a wave pattern that follows the same trend as the wave pattern observed in *Damascus* with a shorter anterior wavelength and a larger total number of waves in the PSM (see also Fig. S4D).

Altogether, these results demonstrate that changes in coupling strength and coupling delays can alter the tissue-level wave pattern observed in the PSM. In particular, it can lead to changes in the anterior wavelength and the number of waves as observed in *Damascus*.

Supplementary References

[1] D. J. Jörg, L. G. Morelli, D. Soroldoni, A. C. Oates, and F. Jülicher. Continuum theory of gene expression waves during vertebrate segmentation. *New J. Phys.* **17**, 093042 (2015)

[2] S. Ares, L. G. Morelli, D. J. Jörg, A. C. Oates, and F. Jülicher. Collective Modes of Coupled Phase Oscillators with Delayed Coupling. *Phys. Rev. Lett.* **108**, 204101 (2012)

[3] D. Soroldoni, D. J. Jörg, L. G. Morelli, D. L. Richmond, J. Schindelin, F. Jülicher, and A. C. Oates. A Doppler effect in embryonic pattern formation. *Science* **345**, 222–225 (2014)

[4] L. G. Morelli, S. Ares, L. Herrgen, C. Schröter, F. Jülicher, and A. C. Oates. Delayed coupling theory of vertebrate segmentation. *HFSP J.* **3**, 55 (2009)

[5] F. Giudicelli, E. M. Ozbudak, G. J. Wright, and J. Lewis. Setting the tempo in development: an investigation of the zebrafish somite clock mechanism. *PLoS Biol.* **5**, e150 (2007)

[6] N. P. Shih, P. François, E. A. Delaune, and S. L. Amacher. Dynamics of the slowing segmentation clock reveal alternating two-segment periodicity. *Development* **142**, 1785–1793 (2015)

[7] M. G. Earl and S. H. Strogatz. Synchronization in oscillator networks with delayed coupling: A stability criterion. *Phys. Rev. E* **67**, 036204 (2003).

[8] L. Herrgen, S. Ares, L. G. Morelli, C. Schröter, F. Jülicher, and A. C. Oates. Intercellular Coupling Regulates the Period of the Segmentation Clock. *Curr. Biol.* **20**, 1244–1253 (2010)
